# Reduction of Hexaazatrinaphthylenes by Masked Divalent Lanthanide Dinitrogen Reagents

**DOI:** 10.1021/acs.inorgchem.5c01681

**Published:** 2025-06-26

**Authors:** Arpan Mondal, Christopher G. T. Price, Alexander Steiner, Jinkui Tang, Richard A. Layfield

**Affiliations:** † Department of Chemistry, School of Life Sciences, 1948 University of Sussex, Brighton BN1 9QJ, U.K.; ‡ Department of Chemistry, University of Liverpool, Crown St, Liverpool L69 7ZD, U.K.; § State Key Laboratory of Rare Earth Resource Utilization, Changchun Institute of Applied Chemistry, 58277 Chinese Academy of Sciences, Changchun 130022, P.R. China

## Abstract

The oxidation state +2 is of interest in rare-earth chemistry since it allows these conventionally redox-inactive metals to be used as reducing agents. However, the divalent oxidation state is difficult to form for most rare-earth elements, and the ensuing compounds are often unstable. Here, we describe an approach to rare-earth reduction chemistry that circumvents the divalent oxidation state by using compounds of trivalent rare earths that store reducing electrons on the dinitrogen ligand [N_2_]^2–^, akin to “masked” divalent reactivity. Thus, the dinitrogen complexes $\left[{\left({\mathrm{Cp}}_{2}^{\mathrm{ttt}}M\right)}_{2}\left(\mu {\mathrm{‐1},\mathrm{2‐N}}_{2}\right)\right]$ (**1**_**M**_, M = Y, Gd, Tb, Dy, Cp^ttt^ = 1,2,4-C_5_^t^Bu_3_H_2_) reduce hexaazatrinaphthylene and its hexamethyl derivative to give trimetallic $\left[{\left({\mathrm{Cp}}_{2}^{\mathrm{ttt}}M\right)}_{3}\left(R_{6}\mathrm{HAN}\right)\right]$ , where the [R_6_HAN]^3–^ ligands (R = H, **2**_**M**_; R = Me, **3**_**M**_) form with *S* = 1/2, and with elimination of N_2_. The structures of **2**_**M**_ and **3**_**M**_ reveal that the *tert*-butyl substituents strongly influence the core geometry of these trimetallic complexes. Analysis of the magnetism and electronic structure of **2**_**Gd**_ and **3**_**Gd**_ identifies ferromagnetic metal-radical exchange, with coupling constants of *J* = +2.87 cm^–1^ and +3.07 cm^–1^, respectively (−2*J* formalism). The unusual ferromagnetic exchange is a consequence of charge transfer to the gadolinium 5d, 6s, and 6p orbitals from the radical ligands.

## Graphical abstract

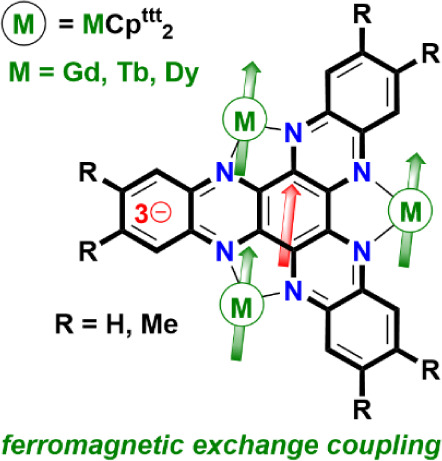

## Introduction

The chemistry of the rare earth elements is dominated by the thermodynamically stable trivalent oxidation state, which is typically redox inactive. The attraction of the more exotic divalent oxidation state in rare-earth chemistry therefore stems partly from its potential use as a one-electron reducing system for a variety of synthetic applications, as shown by the versatility of samarium­(II) reductants in organic chemistry.^1–3^ However, whereas access to the divalent oxidation state is straightforward for the classical three divalent ions of samarium, europium and ytterbium,^4–10^ the same is not true for the other rare-earth elements. Indeed, although the number of molecular neodymium­(II), dysprosium­(II) and thulium­(II) compounds continues to grow,^11–20^ the oxidation state +2 for the full series of rare-earths (with the usual exception of promethium) was established much more recently.^21–31^ Synthetic applications of nonclassical divalent rare-earth compounds have only been developed to a limited extent, which is likely to be a consequence of the sensitivity of these species to the reaction conditions, such as solvent and temperature, and can even depend on the specific element.^31^ The role of alkali metal counter-cations in salt-like divalent compounds may also cause complications.

An indirect solution to the challenge of developing divalent rare-earth reactivity involves storing the reducing electrons on a redox-active ligand bound to a trivalent lanthanide.^32–35^ Here, the aim is to deliver the additional electrons to a substrate, with the redox-active ligand subsequently oxidized and eliminated, and the trivalent lanthanide transferred to the reduced substrate. This approach can be regarded as complementary to sterically induced reduction by bulky tris­(cyclopentadienyl)­lanthanide complexes.^36^ Considering this, we recently reported the trivalent rare-earth dinitrogen complexes $\left[{\left({\mathrm{Cp}}_{2}^{\mathrm{ttt}}M\right)}_{2}\left(\mu {\mathrm{‐1},\mathrm{2‐N}}_{2}\right)\right]$ (**1**_**M**_, *M* = Gd, Tb, Dy; Cp^ttt^ = 1,2,4-C_5_ *^t^* Bu_3_H_2_), in which the reduced ligand [N_2_]^2–^ adopts the end-on coordination mode.^37^ As a proof-of-principle, it was shown that **1**_**M**_ can act as “masked” versions of divalent gadolinium, terbium and dysprosium by transferring one electron per metal to bipyridyl, with the resulting radical anion coordinating to the metal and with N_2_ eliminated. The advantages of this reactivity system are that they can be used in stoichiometric amounts rather than in excess, and they are neutral molecules, meaning that alkali metal ‘ate salts do not complicate matters. With N_2_ as the byproduct, the reactions have an entropic driving force and the purification steps are simpler. In addition, the lutetium version **1**_**Lu**_ was recently reported by Nocton and Simler, and its impressive reactivity toward N_2_ hydrogenation described.^38^

To develop the reactivity of the masked divalent rare-earth dinitrogen compounds, we now focus on the synthesis of radical-bridged multimetallic systems, which have potential applications as molecular magnetic materials such as single-molecule magnets (SMMs).^39–52^ Since most radical-bridged lanthanide molecular magnets feature C_5_Me_5_ (Cp*) as the supporting ligand, using **1**_**M**_ as the reducing agents introduces opportunities to explore how the properties of related systems vary with different cyclopentadienyl substituents. Our interests also extend to masked divalent yttrium, given the suggestion yttrium­(II) may behave differently to similarly sized divalent ions of the late lanthanides.^31^ Thus, using the dinitrogen complexes **1**_**M**_ as the reducing agents, we targeted trimetallic complexes of redox-active hexaazatrinaphthylene (HAN) ligands, which can be reduced with up to three electrons. In the complexes $\left[{\left({\mathrm{Cp}}_{2}^{\mathrm{ttt}}M\right)}_{3}\left(R_{6}\mathrm{HAN}\right)\right]$ , where M = Y, Gd, Tb and Dy, and R = H or CH_3_, the heterocyclic ligands should form as the *S* = 1/2 radical trianions [R_6_HAN]^3–^, facilitating exchange coupling between paramagnetic lanthanides.

## Results and Discussion

First, the new yttrium dinitrogen complex $\left[{\left({\mathrm{Cp}}_{2}^{\mathrm{ttt}}Y\right)}_{2}\left(\mu {\mathrm{‐1},\mathrm{2‐N}}_{2}\right)\right]$ (**1**_**Y**_) was synthesized using the procedure reported for the other **1**_**M**_ complexes.^37^ Adding excess KC_8_ to $\left[{\mathrm{Cp}}_{2}^{\mathrm{ttt}}Y\left({\mathrm{BH}}_{4}\right)\right]$ followed, after 5 days, by N_2_ produced **1**_**Y**_ in an isolated yield of 78%. The target HAN-bridged compounds $\left[{\left({\mathrm{Cp}}_{2}^{\mathrm{ttt}}M\right)}_{3}\left(H_{6}\mathrm{HAN}\right)\right]$ (**2**_**M**_) and $\left[{\left({\mathrm{Cp}}_{2}^{\mathrm{ttt}}M\right)}_{3}\left({\mathrm{Me}}_{6}\mathrm{HAN}\right)\right]$ ­(**3**_**M**_) (M = Y, Gd, Tb, Dy) were then synthesized by adding **1**_**M**_ to each heterocycle in a 3:2 stoichiometry (Scheme 1) (Me_6_HAN = hexamethyl-hexaazatrinaphthylene). The yields are 55–65% for **2**_**M**_ and 64–70% for **3**_**M**_, highlighting an advantage of the masked divalent method since the previously reported complexes [(Cp*_2_M)_3_(H_6_HAN)] (**4**_**M**_) (M = Gd, Tb, Dy), formed by KC_8_ reduction of the heterocycle, were isolated in yields of only 4–6%.^44^

**Scheme 1:**
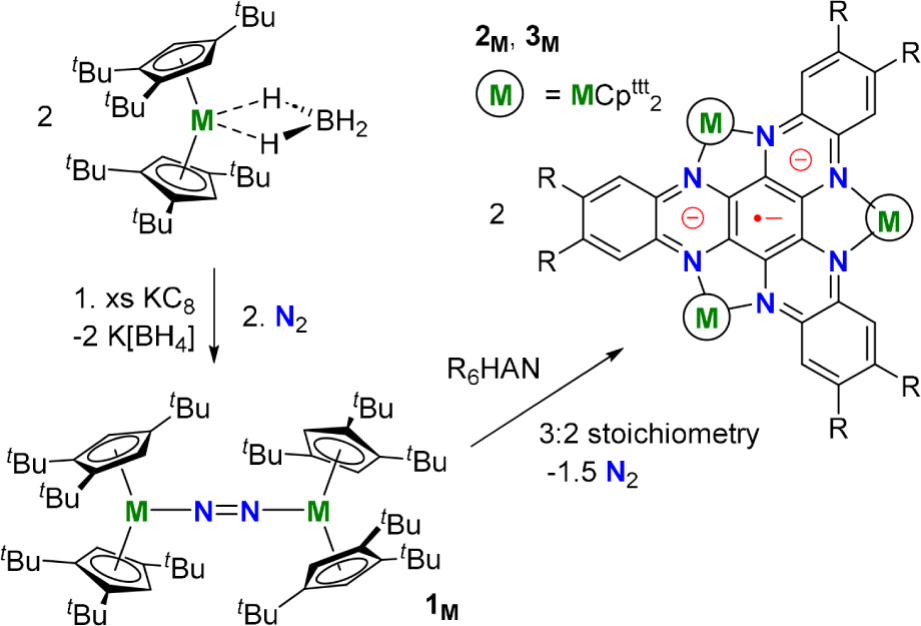
Synthesis of **2**_**M**_ (R = H) and **3**_**M**_ (R = Me). M = Y, Gd, Tb, Dy

The end-on bridging mode of the [N_2_]^2–^ ligand in **1**_**Y**_ was confirmed using X-ray crystallography, which revealed the molecule to be centrosymmetric, with an N1–N1A distance of 1.199 (4) Å and a Y–N distance of 2.258(2) Å (Figure 1, Tables S1 and S2). The Y1···Y1A separation in **1**_**Y**_ is 5.7147(11) Å. The Y– ${\mathrm{Cp}}_{c}^{\mathrm{ttt}}$ distances are 2.3950(12) and 2.4070(12) Å and the ${\mathrm{Cp}}_{c}^{\mathrm{ttt}}$ –Y– ${\mathrm{Cp}}_{c}^{\mathrm{ttt}}$ angle is 143.01(4)°. Overall, the structure of **1**_**Y**_ is comparable to that of the dysprosium congener **1**_**Dy**_,^37^ consistent with the similar size of the two trivalent rare earth cations.^53^

**Figure 1:**
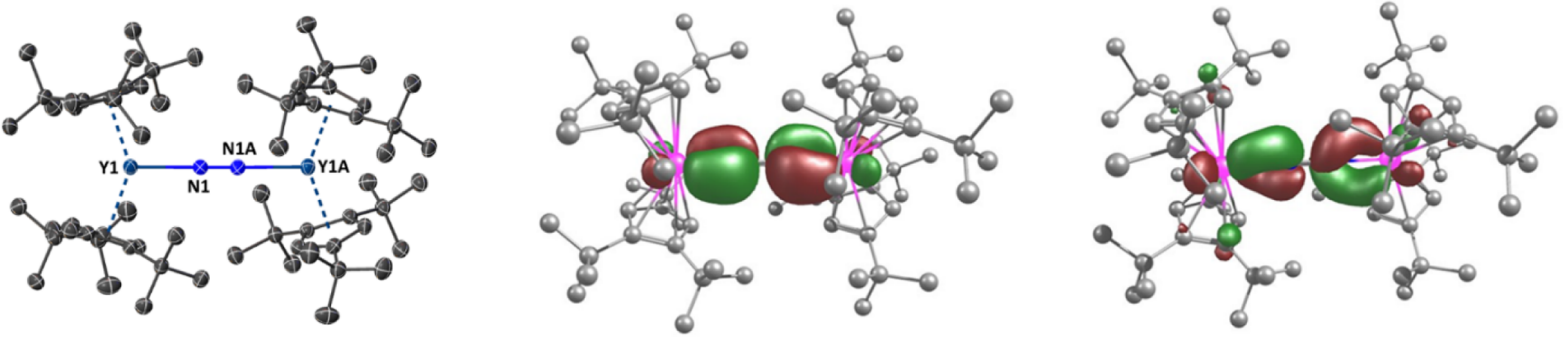
Left: thermal ellipsoid representation (30% probability) of the molecular structure of **1**_**Y**_. Carbon atoms (black) are unlabeled, and hydrogen atoms are not shown. Centre and right: DFT-calculated HOMO and LUMO, respectively, for **1**_**Y**_ (isosurface value = 0.04 a.u.).

The Raman spectrum of **1**_**Y**_ consists of a single sharp absorption at 1630 cm^–1^ corresponding to the N···N stretch (Figures S1 and S2), slightly higher than the value of 1618 cm^–1^ recorded for **1**_**Dy**_, which may be due to the shorter distance within the [N_2_]^2–^ ligand of **1**_**Y**_. Notably, although several yttrium dinitrogen complexes are known, most occur with a side-on [N_2_]^2–^ ligand (as with complexes of this ligand with other rare-earth elements),^9,54–59^ whereas the end-on bridging mode has only been observed before in the complex anion [{(Me_3_Si)_2_N}_3_Y)_2_(μ-1,2-N_2_)]^−^.^31^

Analysis of the bonding in **1**_**Y**_ using DFT (density functional theory) as implemented within ORCA^60^ revealed the highest-occupied molecular orbital (HOMO) and the lowest-unoccupied molecular orbital (LUMO) to be π-type yttrium–nitrogen interactions (Figure 1). The HOMO consists of 33% yttrium 4d character and 50% nitrogen 2p character, and the LUMO consists of 33% yttrium 4d character and 34% nitrogen 2p character. The UV/vis spectrum of **1**_**Y**_ (Figure S10) features a broad absorption centered on 603 nm, which a time-dependent DFT calculation shows is due to transitions from the HOMO to the LUMO+1 and LUMO+2 (630 and 618 nm, respectively), both of which are predominantly yttrium 4d in character (Table S7). Time-dependent DFT calculations also show that a HOMO-to-LUMO+3 transition should occur near 446 nm, although this is obscured in the spectrum by higher-energy transitions within the Cp^ttt^ ligands.

Crystallographic analysis of **2**_**M**_ and **3**_**M**_ revealed individual molecules to be isostructural, consistent with the similarities in their IR spectra (Figures S3–S5) despite differences in their crystal structures. Thus, whereas the novel H_6_HAN-bridged compounds **2**_**Gd**_ and **2**_**Tb**_ crystallize in orthorhombic *Pbcn* and trigonal *R*3, respectively (Figures 2, S6, Tables S3 and S4), previously reported **2**_**Y**_ and **2**_**D** *y*_ crystallize in tetragonal *P*4_1_2_1_2 and trigonal *R*3, respectively.^32^ In contrast, all four Me_6_HAN-bridged compounds **3**_**M**_ crystallize in monoclinic *P*2_1_/*n* (Figures 2, S7–S9, Tables S5 and S6). Compounds **2**_**Tb**_ and **3**_**Tb**_ are used to illustrate the general properties of the series (Figure 2).

**Figure 2:**
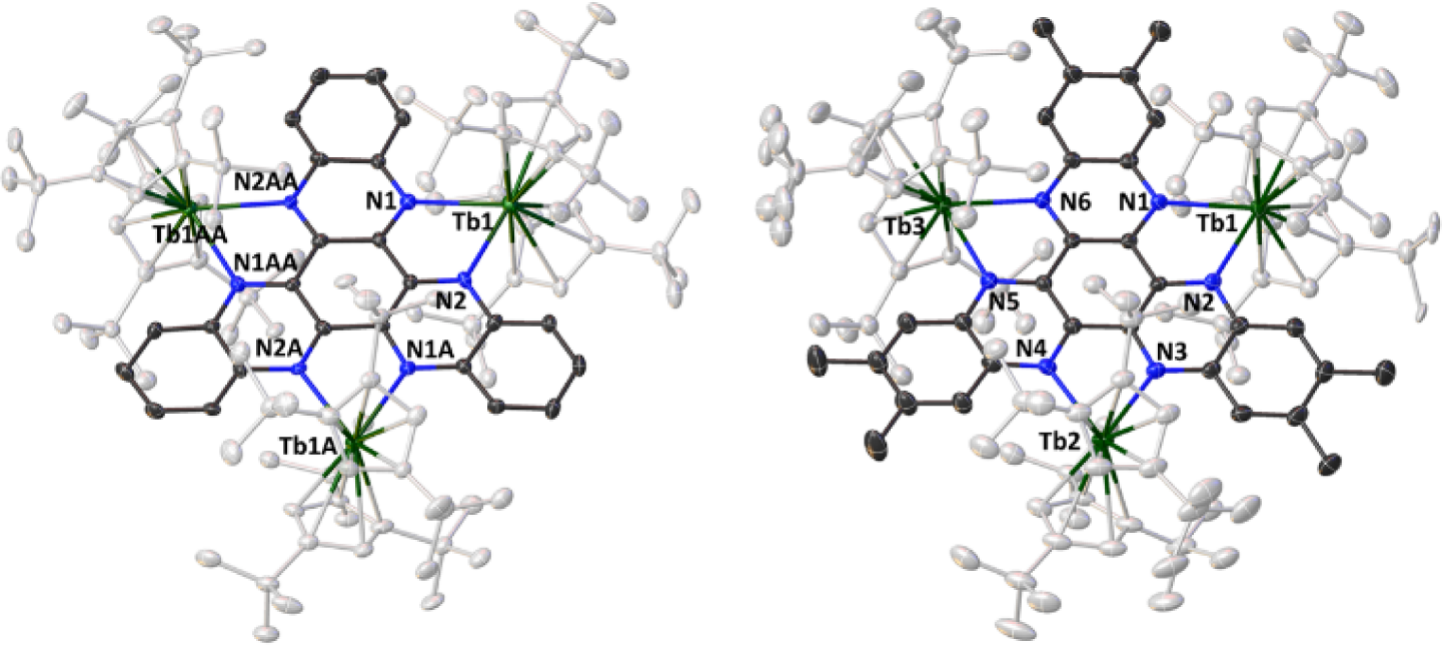
Thermal ellipsoid representation (50% probability) of the molecular structures of **2**_**Tb**_ (left) and **3**_**Tb**_ (right). Carbon atoms (gray, black) are unlabeled, and hydrogen atoms are not shown.

The structures of **2**_**Tb**_ and **3**_**Tb**_ consist of three $\left\{{\mathrm{Cp}}_{2}^{\mathrm{ttt}}\mathrm{Tb}\right\}$ units bridged by the [H_6_HAN]^3–^ and [Me_6_HAN]^3–^ ligands, respectively, the three binding pockets of which adopt the bidentate *N*,*N*-coordination mode. The Tb–N distances involving the symmetry related terbium centers in **2**_**Tb**_ are 2.471(8) and 2.480(10) Å, the Tb–Cp_c_ distances are 2.484(7) and 2.520(7) Å (Cp_c_ denotes the centroid of the Cp^ttt^ ligands), and the Cp_c_-Tb-Cp_c_ angle is 131.1(2)°. In **3**_**Tb**_, the analogous Tb–N distances are in the range 2.458(3)–2.486(3) Å (average 2.470 Å), the Tb–Cp_c_ distances span 2.4973(18)-2.5207(16) Å (average 2.512 Å), and the Cp_c_-Tb-Cp_c_ angles are 129.71(6)–131.25(6)°. The intramolecular Tb···Tb separations in **2**_**Tb**_ and **3**_**Tb**_ are 7.8095(10) Å and 7.8036(9)–7.8431(8) Å, respectively. In **2**_**Tb**_, the C–C and C–N distances in the H_6_HAN ligand are 1.333(18)–1.442(16) Å and 1.323(14)–1.424(15) Å, respectively, and in **3**_**Tb**_ the analogous distances are 1.384(5)–1.443(5) Å and 1.360(4)–1.396(4) Å, respectively, consistent with reduction of the heterocycles to the trianionic form [R_6_HAN]^3–^.^61–63^

As expected, based on the relative radii of the trivalent M^3+^ cations in **2**_**M**_ and **3**_**M**_, the corresponding metal–ligand distances in **2**_**Gd**_ and **3**_**Gd**_ are slightly longer than those in the terbium versions, and those in **2**_**Dy**_/**3**_**Dy**_ and **2**_**Y**_/**3**_**Y**_ are slightly shorter (Tables S4 and S6). Insight into the effect of the *tert*-butyl substituents on the structures of **2**_**M**_ and **3**_**M**_ can be gleaned through a comparison with the structures of the less bulky analogues [(Cp*_2_M)_3_(H_6_HAN)] (**4**_**M**_, M = Tb, Dy); The synthesis of the gadolinium version **4**_**Gd**_ was also reported, but the molecular structure was not determined.^44^ Appreciable differences in key geometric parameters of **2**_**Tb**_ and **3**_**Tb**_ relative to **4**_**Tb**_ are apparent, with a similar pattern found with the dysprosium versions. For example, the Tb···Tb separations in **4**_**Tb**_ are, at 7.6198(11) Å and 7.7163(14) Å, shorter by approximately 0.1–0.2 Å than in **2**_**Tb**_ and **3**_**Tb**_, presumably because the Tb–N distances in **4**_**Tb**_ are also, at 2.395(10)-2.441(10) Å, shorter than those in **2**_**Tb**_ and **3**_**Tb**_. The Tb–Cp_c_ distances of 2.383(14)-2.422(7) Å in **4**_**Tb**_ are shorter by about 0.1 Å than the equivalent distances in **2**_**Tb**_ and **3**_**Tb**_. Overall, complex **4**_**Tb**_ has a more compact coordination environment than **2**_**Tb**_ and **3**_**Tb**_. The longer bond lengths in **2**_**Tb**_ and **3**_**Tb**_ are, therefore, most likely a consequence of intramolecular steric repulsion between the *tert*-butyl groups. The significant structural differences observed in the terbium complexes would presumably also be present in the equivalent gadolinium complexes, potentially impacting on the magnetic exchange interactions, which was explored using EPR spectroscopy and magnetic measurements.

## Magnetic Properties

The X-band EPR spectra of **3**_**Y**_, **2**_**Gd**_ and **3**_**Gd**_ were measured in the solid state at 298 K (Figures S11–S13) and found to consist of singlet resonances with isotropic *g*-values of 2.009, 2.007 and 2.009, respectively. The line width peak-to-peak (LWPP) of the EPR signal of **3**_**Y**_ is 0.049 mT, similar to that of 0.065 mT reported for **2**_**Y**_^32^ and noticeably smaller than the LWPP values of 1.271 mT and 1.558 mT for **2**_**Gd**_ and **3**_**Gd**_, respectively. The broader EPR signals for the gadolinium complexes are likely due to exchange interactions, which was investigated further with magnetic susceptibility measurements.

The molar magnetic susceptibility $\left(\chi_{M}\right)$ was determined for **2**_**Gd**_ and **3**_**Gd**_ in the temperature range 2-300 K using an applied field of 1000 Oe. Curie-Weiss plots of $\chi_{M}^{-1}$ versus temperature for both compounds are linear in the region 25-300 K before showing slight upward curvature at lower temperatures (Figure S14). A linear fit of the plots using the Curie-Weiss formula $\chi_{M}^{-1}=\left(T-\theta \right)/C$ , where θ and *C* are the Weiss and Curie constants, respectively, yielded θ= +5.32 K and C= 23.72 cm^3^ K^–1^ mol^–1^ for **2**_**Gd**_ and θ= +6.05 K and C= 24.10 cm^3^ K^–1^ mol^–1^ for **3**_**Gd**_. The positive Weiss constants determined for **2**_**Gd**_ and **3**_**Gd**_ indicate ferromagnetic exchange between the gadolinium centers and the *S* = 1/2 [R_6_HAN]^3–^ ligands.

To quantify the exchange interactions, $\chi_{M}T$ was plotted as a function of temperature (Figure 3) and a fit of the data obtained using the spin Hamiltonian in eq 1, where J is the exchange coupling constant, $S_{\mathrm{Gd}}$ denotes a spin of 7/2 on each Gd^3+^ center, $S_{\mathrm{rad}}$ denotes a spin of 1/2 on the [HAN]^3–^ ligand, β is the Bohr magneton, and *B* is the magnetic field.

1 $$\begin{array}{l} Ĥ=-2J\left(S_{\mathrm{rad}}\cdot S_{\mathrm{Gd}1}+S_{\mathrm{rad}}\cdot S_{\mathrm{Gd}2}+S_{\mathrm{rad}}\cdot S_{\mathrm{Gd}3}\right)\\ +\beta \left(g_{\mathrm{Gd}1}\cdot S_{\mathrm{Gd}1}+g_{\mathrm{Gd}2}\cdot S_{\mathrm{Gd}2}+g_{\mathrm{Gd}3}\cdot S_{\mathrm{Gd}3}+g_{\mathrm{rad}}\cdot S_{\mathrm{rad}}\right)\cdot B \end{array}$$

**Figure 3:**
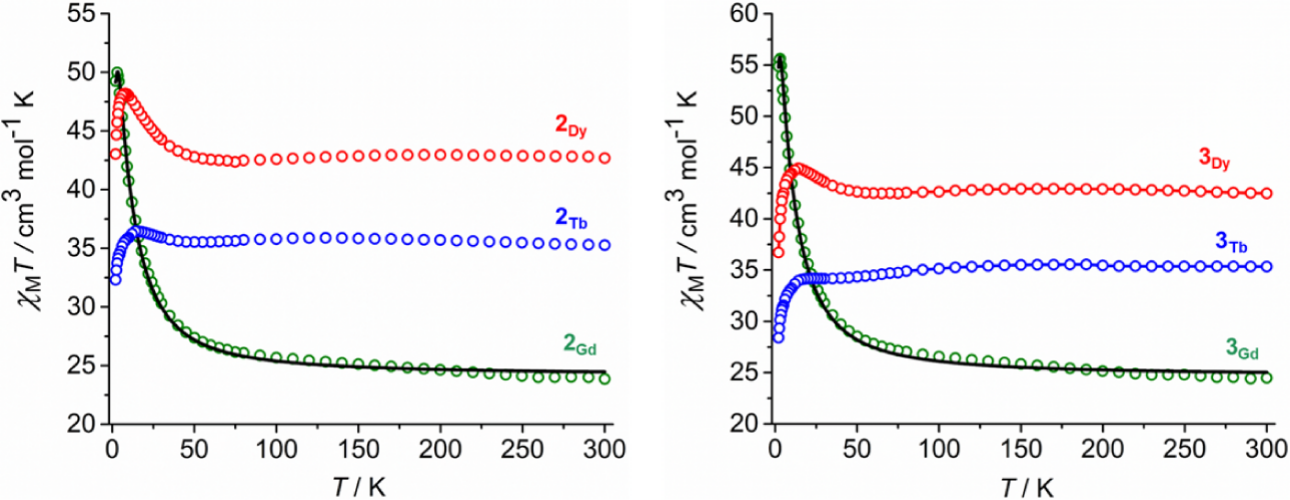
Plots of $\chi_{M}T\left(T\right)$ for **2**_**M**_ (left) and **3**_**M**_ (right) (*M* = Gd, Td, Dy) in an applied field of 1000 Oe. Solid black lines for **2**_**Gd**_ and **3**_**Gd**_ represent fits to the data using the parameters stated in the text. Data for **2**_**Dy**_ are reproduced from ref 32.

The $\chi_{M}T\left(T\right)$ plots for **2**_**Gd**_ and **3**_**Gd**_ are similar (Figure 3), with the $\chi_{M}T$ values of 23.82 cm^3^ K mol^–1^ and 24.06 cm^3^ K mol^–1^, respectively, at 300 K being close to the theoretical value of 24.0 cm^3^ K mol^–1^ for three uncoupled Gd^3+^ ions and an *S* = 1/2 radical.^64^ On lowering the temperature, a gradual rise in $\chi_{M}T$ occurs before a steeper increase in around 50 K. At 2 K, $\chi_{M}T$ values of 49.23 and 56.22 cm^3^ K mol^–1^ are reached for **2**_**Gd**_ and **3**_**Gd**_, respectively. Good fits of the susceptibility data using eq 1 with $g_{\mathrm{Gd}}=g_{\mathrm{rad}}=2.0$ and small intermolecular exchange terms ( zJ= −0.002, −0.001 cm^–1^, Table S8) yielded coupling constants for the gadolinium-radical exchange of J= +2.87 ± 0.03 cm^–1^ for **2**_**Gd**_ and J= +3.07 ± 0.04 cm^–1^ for **3**_**Gd**_. The same model gives good fits of the isothermal field dependence of the magnetization, *M*(*H*) at 2, 3, and 5 K, with saturation magnetization values of 22.17 Nβ and 22.14 Nβ at 2 K and 7 T (Figures S15 and S19), both of which are close to the expected value of 22 Nβ.

For **2**_**Tb**_ and **3**_**Tb**_, the $\chi_{M}T\left(T\right)$ data indicate similar exchange properties to those observed for the gadolinium analogues, as do the data for previously reported **2**_**Dy**_ and the novel compound **3**_**Dy**_ (Figures 3, S16, S20 and S23). For **2**_**Tb**_, the value of $\chi_{M}T$ at 300 K is 36.46 cm^3^ K mol^–1^ and for **3**_**Tb**_ it is 35.34 cm^3^ K mol^–1^, both of which are close to the theoretical value of 35.84 cm^3^ K mol^–1^ for three uncoupled Tb^3+^ ions and an *S* = 1/2 radical.^64^ For **2**_**Tb**_, a slight increase in $\chi_{M}T$ to 36.46 cm^3^ K mol^–1^ occurs at 14 K, with a smaller increase to 34.44 cm^3^ K mol^–1^ observed for **3**_**Tb**_ at 15 K. The value of $\chi_{M}T$ then decreases to 32.31 cm^3^ K mol^–1^ for **2**_**Tb**_ and to 28.40 cm^3^ K mol^–1^ for **3**_**Tb**_ at 2 K. A similar $\chi_{M}T\left(T\right)$ profile is observed for **3**_**Dy**_, with susceptibility values of 42.48, 44.49, and 36.71 cm^3^ K mol^–1^ at 300, 14, and 2 K, respectively, with the value at 300 K matching well to the theoretical value of 42.89 cm^3^ K mol^–1^ for three Dy^3+^ ions and the radical ligand.

Since several multimetallic radical-bridged terbium and dysprosium complexes show SMM behavior, the AC magnetic susceptibility and magnetic hysteresis properties of **2**_**Tb**_, **3**_**Tb**_ and **3**_**Dy**_ were studied. In zero DC field, no maxima were observed for any of the compounds in the frequency dependence of the imaginary component of the AC susceptibility (Figures S21, S23 and S24), and no *M*(*H*) hysteresis loops were observed at 2 K (Figures S18, S22, S25). Similar properties were previously found for **2**_**Dy**_. Hence, in all four compounds, the dominant relaxation mechanism is quantum tunneling of the magnetization (QTM). The absence of SMM behavior in **2**_**Tb**_, **3**_**Tb**_, and **2**_**Dy**_ and **3**_**Dy**_ may seem surprising considering hysteresis loops occurring up to 3.5 K for **4**_**Dy**_, and Raman relaxation occurring for **4**_**Tb**_. However, based on the axiality model developed for dysprosium and other “oblate” lanthanide SMMs,^32^ the significantly longer M–Cp_c_ distances and smaller metallocene bending angles in the Cp^ttt^-ligated complexes should result in a weaker and less-axial crystal field, consistent with the absence of SMM behavior.

Considering that lanthanide-radical exchange interactions involving *N*-heterocycles are typically antiferromagnetic,^43,50,65^ the ferromagnetic exchange in **2**_**Gd**_ and **3**_**Gd**_ is remarkable. Furthermore, the magnitude of the *J*-values determined for the gadolinium compounds is substantially larger than a typical exchange coupling constant found in gadolinium complexes of diamagnetic ligands. Indeed, the *J*-values for **2**_**Gd**_ and **3**_**Gd**_ are similar in magnitude to the equivalent parameter of −5.0 cm^–1^ reported for **4**_**Gd**_ (−2*J* formalism),^44^ but the switch from antiferromagnetic to ferromagnetic is surprising, a phenomenon that we sought to explain with the aid of theoretical calculations.

## Theoretical Studies

To gain insight into the electronic structure and the exchange interactions, we performed DFT calculations and natural bond orbital (NBO) analyses on **3**_**Y**_, **2**_**Gd**_ and **3**_**Gd**_ using ORCA.^60^ In agreement with the presence of one unpaired electron in **3**_**Y**_, the doublet state of the [Me_6_HAN]^3–^ ligand is calculated to be more stable than the quartet by 30.6 kJ mol^–1^ (Table S9). For all three compounds, the SOMO (singly occupied molecular orbital), SOMO–1 and LUMO correspond to the π* orbitals of the trianionic heterocyclic ligands, whereas the LUMO+1, LUMO+2, and LUMO+3 orbitals are largely metal d-orbital in character (Figures 4 and S26–S28).

**Figure 4:**
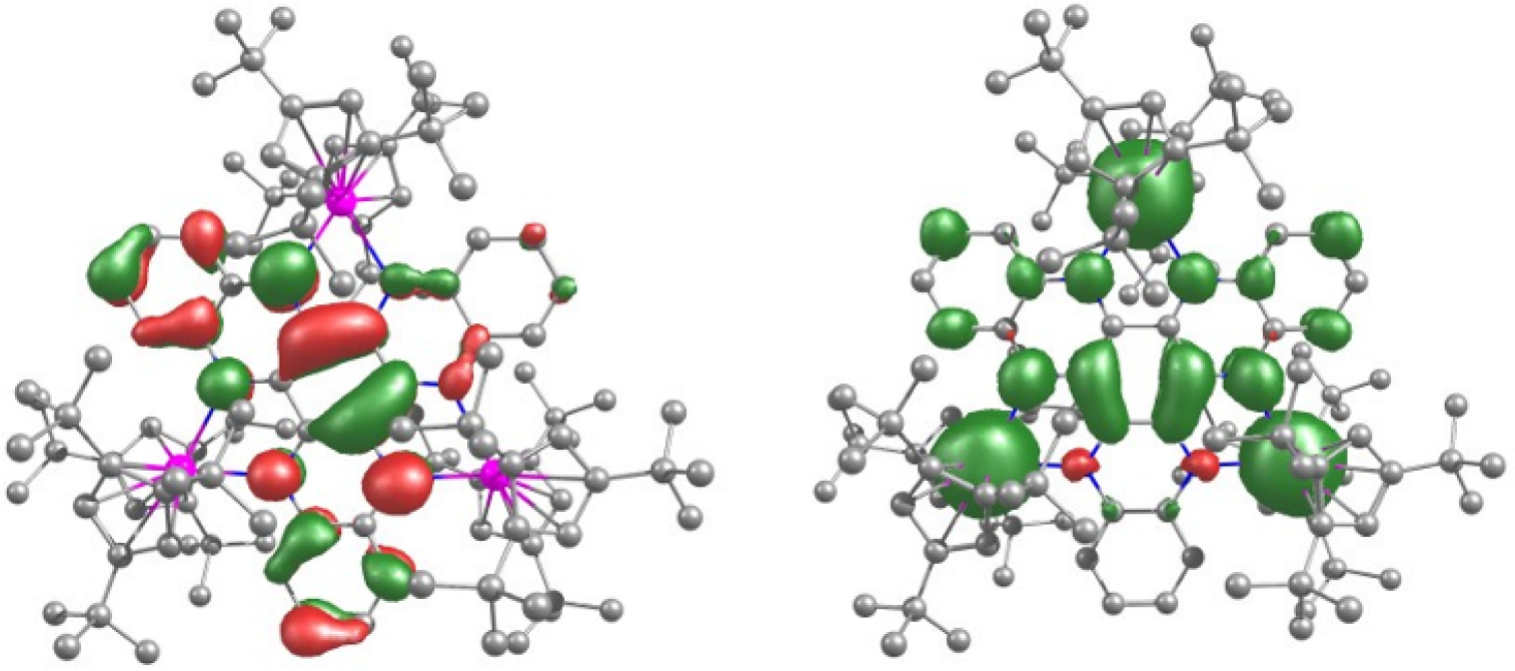
SOMO (left, isosurface value 0.027 a.u.) and spin density plot (right, isosurface value 0.0015 a.u.) for **2**_**Gd**_. Hydrogen atoms not shown.

The spin density plot for the doublet state of **3**_**Y**_ indicates that the radical electron is delocalized extensively across the [Me_6_HAN]^3–^ ligand, with significant spin density on the nitrogen atoms and on the carbon atoms of the central C_6_ ring (Figure S29 and Table S10). The spin density analysis of **2**_**Gd**_ and **3**_**Gd**_ shows a similar picture, with delocalization of the radical across the heterocyclic ligand and with spherical spin density around each gadolinium (Figures 4 and S29). The spin density values of 7.107–7.098 for the metal centers in **2**_**Gd**_ and **3**_**Gd**_ (Table S10) are slightly higher than the expected value for a 4f^7^ ion because of spin polarization effects, where each gadolinium gains spin density from the nitrogen atoms. This effect is consistent with the observation of strong metal–ligand exchange interactions in **2**_**Gd**_ and **3**_**Gd**_.

The NBO analysis of **2**_**Gd**_ and **3**_**Gd**_ provides further insight into the exchange mechanism. Usually, the net (observed) exchange interaction arises from a combination of the antiferromagnetic and ferromagnetic components, both of which are mainly controlled by the overlap between the magnetic orbitals of the metal and the π* orbitals of the radical ligand. However, it has been shown for several series of gadolinium-3d metal and gadolinium radical complexes that empty gadolinium 5d/6s/6p orbitals also play an important role, thought to involve charge transfer from the radical to the metal that results in partial occupation of these higher-lying orbitals.^66–71^ Charge-transfer interactions of this type contribute to ferromagnetic exchange. In line with this model, the NBO second-order perturbation analysis of **2**_**Gd**_ and **3**_**Gd**_ reveals significant donor–acceptor interactions between the nitrogen 2p orbitals and the empty gadolinium 5d/6s/6p orbitals (Tables S11 and S12). The calculated stabilization energies for the interactions in **2**_**Gd**_ and **3**_**Gd**_ are similar, lying in the range 19.3–55.8 kJ mol^–1^ and 30.8–47.2 kJ mol^–1^, respectively, consistent with a ferromagnetic contribution for both complexes. These stabilization energies are comparable in magnitude to those calculated for several ferromagnetically coupled gadolinium complexes with nitroxide radical ligands.^66^

The differences in the primary and secondary coordination environments of the gadolinium centers in **2**_**Gd**_ and **3**_**Gd**_ relative to those in **4**_**Gd**_ are evidently responsible for the switch to ferromagnetic exchange. Our results demonstrate that even simple changes to the peripheral Cp ligand substituents in radical-bridged lanthanide complexes can have a major effect on the magnetic exchange coupling. This “steric” approach to modifying the magnetism is complementary to other substituent-based approaches in which electronic (inductive, mesomeric) effects can also be used to tune the magnetic properties.^48^ Since many HAN-type ligands with a variety of electron-releasing or withdrawing substituents are accessible, it should now be possible to tune the magnetic properties of lanthanide complexes of radical HAN ligands using a combination of electronic and steric handles. Given the ease with which substituents on cyclopentadienyl ligands can be changed, the steric aspect could be enabled using lanthanide dinitrogen complexes, based on **1**_**M**_, as masked divalent reducing agents.

## Conclusions

The new yttrium dinitrogen complex $\left[{\left({\mathrm{Cp}}_{2}^{\mathrm{ttt}}Y\right)}_{2}\left(\mu {\mathrm{‐1},\mathrm{2‐N}}_{2}\right)\right]$ (**1**_**Y**_) and the previously reported versions **1**_**M**_ (*M* = Gd, Tb, Dy) reduce hexaazatrinaphthylene and its hexamethyl derivative to their trianionic forms, producing trimetallic $\left[{\left({\mathrm{Cp}}_{2}^{\mathrm{ttt}}M\right)}_{3}\left(H_{6}\mathrm{HAN}\right)\right]$ (**2**_**M**_) and $\left[{\left({\mathrm{Cp}}_{2}^{\mathrm{ttt}}M\right)}_{3}\left({\mathrm{Me}}_{6}\mathrm{HAN}\right)\right]$ (**3**_**M**_) (M = Y, Gd, Tb, Dy), respectively, in good yields. Steric interactions between the *tert*-butyl substituents in **2**_**M**_ and **3**_**M**_ lead to longer metal-Cp^ttt^ and metal–nitrogen distances than found in the related, less bulky trimetallic complexes [(Cp*_2_M)_3_(H_6_HAN)] (**4**_**M**_, M = Tb, Dy). The elongated metal-Cp^ttt^ interactions generate a weaker axial crystal field, explaining why the terbium and dysprosium complexes do not show SMM behavior. EPR spectroscopy and magnetic measurements on **2**_**M**_ and **3**_**M**_ reveal that the [R_6_HAN]^3–^ ligands are present as their *S* = 1/2 forms, which is reinforced through a DFT study of **3**_**Y**_. Analysis of the magnetic susceptibility of **2**_**Gd**_ and **3**_**Gd**_ produces the striking observation of ferromagnetic metal-radical exchange, with coupling constants of J= +2.87 cm^–1^ and J= +3.07 cm^–1^, respectively (−2*J* formalism). A DFT study of the relevant magnetic orbitals and spin density revealed extensive delocalization of unpaired spin across the [R_6_HAN]^3–^ ligands and the Gd^3+^ centers. Charge transfer from the radical ligand to the higher-lying metal 5d/6s/6p orbitals appears to be responsible for the ferromagnetic exchange.

Considering our findings on **2**_**M**_ and **3**_**M**_, our future work on radical-ligated lanthanide complexes will use the masked divalent lanthanide methodology to explore how exchange interactions and other magnetic phenomena can be modified through changes to the secondary coordination sphere.

## Experimental Section

Synthesis methods for the compounds reported in this article are provided below. No uncommon hazards are noted. General protocols, spectroscopic, crystallographic and magnetic measurement details, along with computational methods, are described in the Supporting Information.

### Synthesis of [{(Cp^ttt^)_2_Y}_2_(μ-1,2-N_2_)] (**1_Y_**)

Hexane (20 mL) was added to a mixture of (Cp^ttt^)_2_Y­(BH_4_) (200 mg, 0.350 mmol) and KC_8_ (237 mg, 1.75 mmol) at room temperature and the resulting suspension was stirred for 5 days, during which time an intense orange-colored solution developed. The solution was filtered and the filtrate freeze–pump–thaw degassed, and then an excess of N_2_ gas was admitted at atmospheric pressure, which resulted in the immediate formation of a deep blue solution. The mixture was stored at −45 °C overnight to effect complete precipitation of the product, which was isolated by filtration and dried under vacuum to yield **1**_**Y**_ as a blue microcrystalline solid (155 mg, 78% based on yttrium). Elemental analysis (%), found (calculated) for C_68_H_116_Y_2_N_2_: C 71.49 (71.68); H 10.18 (10.26); N 2.35 (2.46). FTIR ( υ̃ /cm^–1^): 3000–2800 (m, br, C–H), 1489–1456 (m, br), 1388 (s, str.), 1358 (s, str.), 1241 (s, str.), 1000 (s, str.), 830 (s, str.), 774 (s, str.). Raman ( υ̃ /cm^–1^): 1630 cm^–1^ (N_2_ stretch). Note: compound **1**_**Y**_ has poor solubility in hydrocarbon and aromatic solvents at room temperature and appears to decompose upon heating. Consequently, it was not possible to acquire ^1^H NMR data on this compound.

### Synthesis of [{(Cp^ttt^)_2_Gd}_3_(HAN)]·5­(Benzene) (**2_Gd_** ·5 Benzene)

Toluene (6 mL) was added to a mixture of **1**_**Gd**_ (100 mg, 0.078 mmol) and HAN (20 mg, 0.052 mmol), and the suspension was stirred for 24 h, producing a deep-brown solution. The solvent was then removed under reduced pressure to give a solid residue, which was washed with hexane and redissolved in the minimum amount of hot THF (5 mL). Deep brown microcrystals formed during this process. Storing the solution at −45 °C for 3 days led to the formation of more deep brown microcrystals of **2**_**Gd**_ (75 mg, 64% based on gadolinium). Crystals suitable for single crystal X-ray diffraction were grown by slow evaporation of a benzene/hexane mixed solution. Elemental analysis (%), found (calculated) for C_126_H_186_N_6_Gd_3_ (without solvent): C 66.98 (67.06); H 8.29 (8.31); N 3.69 (3.72). FTIR ( υ̃ /cm^–1^): 3000–2800 (m, br, C–H), 1556 (s, w), 1462 (s, w), 1414–1317 (m, br), 1239 (s, str.), 1072 (s, str.), 831 (s, str.), 779 (s, str.), 618 (s, str.).

### Synthesis of [{(Cp^ttt^)_2_Tb}_3_(HAN)]·2.1­(THF)·0.3­(Toluene) (**2_Tb_** ·2.1 THF·0.3 Toluene)

Compound **2**_**Tb**_ was synthesized using the procedure described for **2**_**Gd**_, using **1**_**Tb**_ (100 mg, 0.078 mmol) and HAN (20 mg, 0.052 mmol), and isolated as deep brown microcrystals (72 mg, 62% based on terbium). Crystals suitable for single-crystal X-ray diffraction were grown from the THF and toluene mixture solution at −35 °C. Elemental analysis (%), found (calculated) for C_126_H_186_N_6_Tb_3_ (without solvent): C 66.80 (66.91); H 8.24 (8.29); N 3.68 (3.72). FTIR ( υ̃ /cm^–1^): 3000–2800 (m, br, C–H), 1558 (s, w), 1461 (s, w), 1413–1315 (m, br), 1237 (s, str.), 1070 (s, str.), 830 (s, str.), 778 (s, str.), 618 (s, str.).

### Synthesis of [{(Cp^ttt^)_2_Dy}_3_(HAN)] (**2_Dy_**)

Compound **2**_**Dy**_ was synthesized using the procedure described for **2**_**Gd**_, using **1**_**Dy**_ (100 mg, 0.078 mmol) and HAN (20 mg, 0.052 mmol), and isolated as deep brown microcrystals (77 mg, 65% based on dysprosium). Crystals suitable for single crystal X-ray diffraction were grown by slow evaporation of a benzene/hexane solution. Elemental analysis (%), found (calculated) for C_126_H_186_N_6_Dy_3_: C 66.80 (66.60); H 8.24 (8.25); N 3.68 (3.70). FTIR ( υ̃ /cm^–1^): absorptions match those reported previously.^32^

### Synthesis of [{(Cp^ttt^)_2_Y}_3_(HAN)] (**2_Y_**)

Compound **2**_**Y**_ was synthesized using the procedure described for **2**_**Gd**_, using **1**_**Y**_ (100 mg, 0.087 mmol) and HAN (22.3 mg, 0.058 mmol), and isolated as deep brown microcrystals (65 mg, 55% based on yttrium). Crystals suitable for single crystal X-ray diffraction were grown by slow evaporation of a benzene/hexane solution. Elemental analysis (%), found (calculated) for C_126_H_186_N_6_Y_3_: C 73.56 (73.76); H 8.99 (9.14); N 4.03 (4.10). FTIR ( υ̃ /cm^–1^): absorptions match those reported previously.^32^

### Synthesis of [{(Cp^ttt^)_2_Y}_3_(Me_6_HAN)]·2.2­(Toluene) (**3_Y_** ·2.2 Toluene)

Toluene (8 mL) was added to a mixture of **1**_**Y**_ (60 mg, 0.052 mmol) and Me_6_HAN (16 mg, 0.034 mmol), and the suspension was stirred for 24 h, producing a deep-brown solution and a precipitate. The solvent was then removed under reduced pressure to give a solid residue, which was redissolved in the minimum amount of hot toluene (5 mL). Storing the solution at −45 °C for a 3 days led to the formation of deep brown crystals of **3**_**Y**_ (50 mg, 67% based on yttrium) which were suitable for single-crystal X-ray diffraction. Elemental analysis (%), found (calculated) for C_132_H_198_N_6_Y_3_ (without solvent): C 74.10 (74.23); H 9.29 (9.34); N 3.89 (3.93). FTIR ( υ̃ /cm^–1^): 3000–2800 (m, br, C–H), 1538 (s, w), 1458 (s, w), 1388–1317 (m, br), 1260–1238 (d, str.), 1096–1080 (d, str.), 825–809 (d, str.), 632–618 (d, str.).

### Synthesis of [{(Cp^ttt^)_2_Gd}_3_(Me_6_HAN)]·2.5­(Toluene) (**3_Gd_** ·2.5 Toluene)

Compound **3**_**Gd**_ was synthesized using the procedure described for **3**_**Y**_, using **1**_**Gd**_ (100 mg, 0.078 mmol) and Me_6_HAN (24.4 mg, 0.052 mmol), and isolated as deep brown crystals (80 mg, 65% based on gadolinium). Elemental analysis (%), found (calculated) for C_132_H_198_N_6_Gd_3_ (without solvent): C 67.40 (67.73); H 8.44 (8.53); N 3.48 (3.59). FTIR ( υ̃ /cm^–1^): 3000–2800 (m, br, C–H), 1537 (s, w), 1458 (s, w), 1388–1311 (m, br), 1259–1235 (d, str.), 1096–1080 (d, str.), 825–809 (d, str.), 631–619 (d, str.).

### Synthesis of [{(Cp^ttt^)_2_Tb}_3_(Me_6_HAN)]·4.5­(Toluene) (**3_Tb_** ·4.5 Toluene)

Compound **3**_**Tb**_ was synthesized using the procedure described for **3**_**Y**_, using **1**_**Tb**_ (100 mg, 0.078 mmol) and Me_6_HAN (24.4 mg, 0.052 mmol), and isolated as deep brown crystals (78 mg, 64% based on terbium). Elemental analysis (%), found (calculated) for C_132_H_198_N_6_Tb_3_ (without solvent): C 67.62 (67.59); H 8.51 (8.51); N 3.56 (3.58). FTIR ( υ̃ /cm^–1^): 3000–2800 (m, br, C–H), 1537 (s, w), 1458 (s, w), 1389–1311 (m, br), 1259–1236 (d, str.), 1095–1079 (d, str.), 823–808 (d, str.), 631–620 (d, str.).

### Synthesis of [{(Cp^ttt^)_2_Dy}_3_(Me_6_HAN)]·2.5­(Toluene) (**3_Dy_**)

Compound **3**_**Dy**_ was synthesized using the procedure described for **3**_**Y**_, using **1**_**Dy**_ (100 mg, 0.077 mmol) and Me_6_HAN (24.2 mg, 0.051 mmol), and isolated as deep brown crystals (85 mg, 70% based on dysprosium). Elemental analysis (%), found (calculated) for C_132_H_198_N_6_Dy_3_ (without solvent): C 67.10 (67.28); H 8.42 (8.47); N 3.53 (3.57). FTIR ( υ̃ /cm^–1^): 3000–2800 (m, br, C–H), 1537 (s, w), 1458 (s, w), 1387–1311 (m, br), 1258–1235 (d, str.), 1095–1079 (d, str.), 824–810 (d, str.), 632–621 (d, str.).

## Supplementary Material



## Data Availability

Other data that support the findings of this study are openly available at DOI: 10.25377/sussex.28615223.

## Notes

Synthesis, spectroscopic characterization, crystallographic details, magnetic measurements, and computational details (PDF)
