# Supplementary material for: Reduction of Hexaazatrinaphthylenes by Masked Divalent Lanthanide Dinitrogen Reagents
Source: Inorg Chem. 2025 Jun 26;64(26):13309–17. doi: 10.1021/acs.inorgchem.5c01681 (PMC12239087; doi:10.1021/acs.inorgchem.5c01681)
Supplement: Supplementary file 1 [file ic5c01681_si_001.pdf]

Supporting Information

Reduction of Hexaazatrinaphthylenes by Masked Divalent Lanthanide Dinitrogen Reagents

Arpan Mondal,[a] Christopher G. T. Price,[a] Alexander Steiner,[b] Jinkui Tang,[c] and Richard A. Layfield*[a]

a Department of Chemistry, School of Life Sciences, University of Sussex, Brighton, BN1 9QJ, U.K. r.layfield@sussex.ac.uk

b Department of Chemistry, University of Liverpool, Crown St, Liverpool L69 7ZD, U.K.

c State Key Laboratory of Rare Earth Resource Utilization, Changchun Institute of Applied Chemistry, Chinese Academy of Sciences, Changchun, 130022, P.R. China.

Contents

General considerations S1
Raman and IR Spectra S2-S6
X-Ray Crystallography S7-S16
DFT Calculations on $\mathbf{1}_\mathbf{Y}$ S17-S18
EPR Spectroscopy S19-S21
Magnetic Property Measurements S22-S30
DFT Calculations on $\mathbf{3}_\mathbf{Y}$, $\mathbf{2}_\mathbf{Gd}$ and $\mathbf{3}_\mathbf{Gd}$ S31-S34
References S35-S36

General considerations

All reactions were carried out using rigorous anaerobic, anhydrous conditions using argon or nitrogen atmospheres and Schlenk/glovebox techniques. Solvents were refluxed over an appropriate drying agent for at least three days (molten potassium for toluene, Na/K alloy for hexane), and then distilled and degassed via three freeze-pump-thaw cycles. Solvents were then stored in ampoules over potassium mirrors. Literature procedures were used to synthesise [Ln(BH_4)$_3$(THF)$_3$] (Ln = Y, Gd, Tb, Dy),[1] $Cp^{ttt}H$,[2] [(Cp^{ttt})$_2$Ln(BH_4)],[3] [{(Cp^{ttt})$_2$M}$_2$(μ-1,2-N_2)] ($\mathbf{1}_\mathbf{Ln}$),[4] hexaazatrinaphthylene (HAN) and hexamethylhexaazatrinaphthylene (Me_6HAN).[5] KCp^{ttt} was synthesized by reacting $Cp^{ttt}H$ with KN($SiMe_3$)$_2$ in toluene overnight. No uncommon hazards are noted. Elemental analyses were conducted by Elemental Microanalysis Ltd. FTIR spectra were recorded on a Bruker Alpha spectrometer with a platinum-diamond ATR module, housed in a glovebox. Raman spectra were measured using a Renishaw inVia confocal Raman microscope using an excitation laser wavelength of 532 nm and 1800 mm^{-1} grating. Laser power was kept below 0.5 mW to reduce heating effects. Measurements used a $\times$20 magnification objective, resulting in a laser spot size of about 1.60 mm. Spectra were baseline corrected, and cosmic rays were removed using WiRE software. For the baseline, the software automatically uses an ‘intelligent fitting’, which excludes regions with peaks and fits the rest of the spectrum using a polynomial expression. EPR spectra were recorded at the PEPR Facility at Imperial College London. Bruker EMX spectrometer was used in CW mode equipped with a Bruker ER049X SuperX microwave bridge, a Bruker ER4122SHQE resonator, and an Oxford Instruments ITC503 temperature controller.

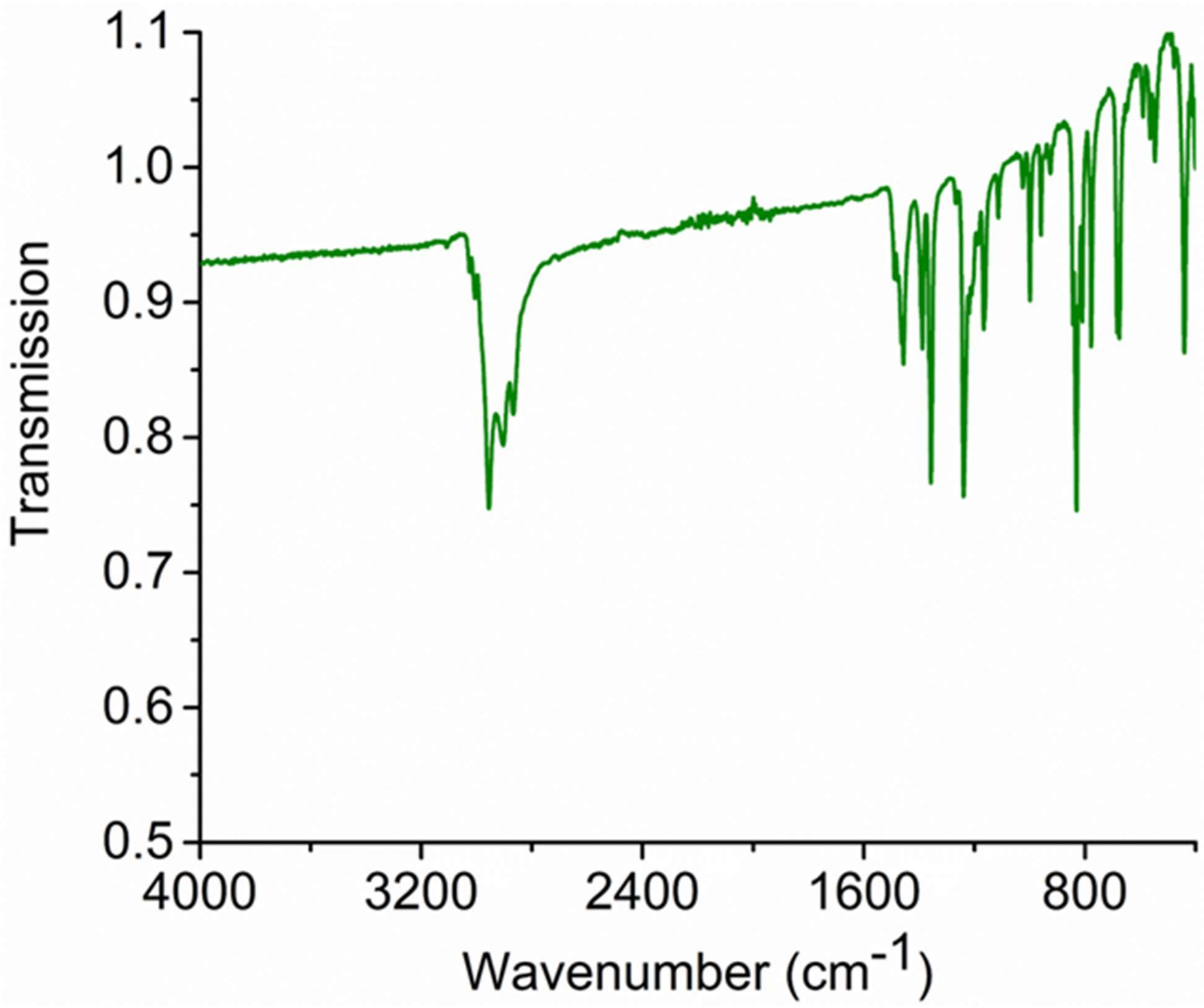

Figure S1. FTIR spectrum of $\mathbf{1}_{Y}$.

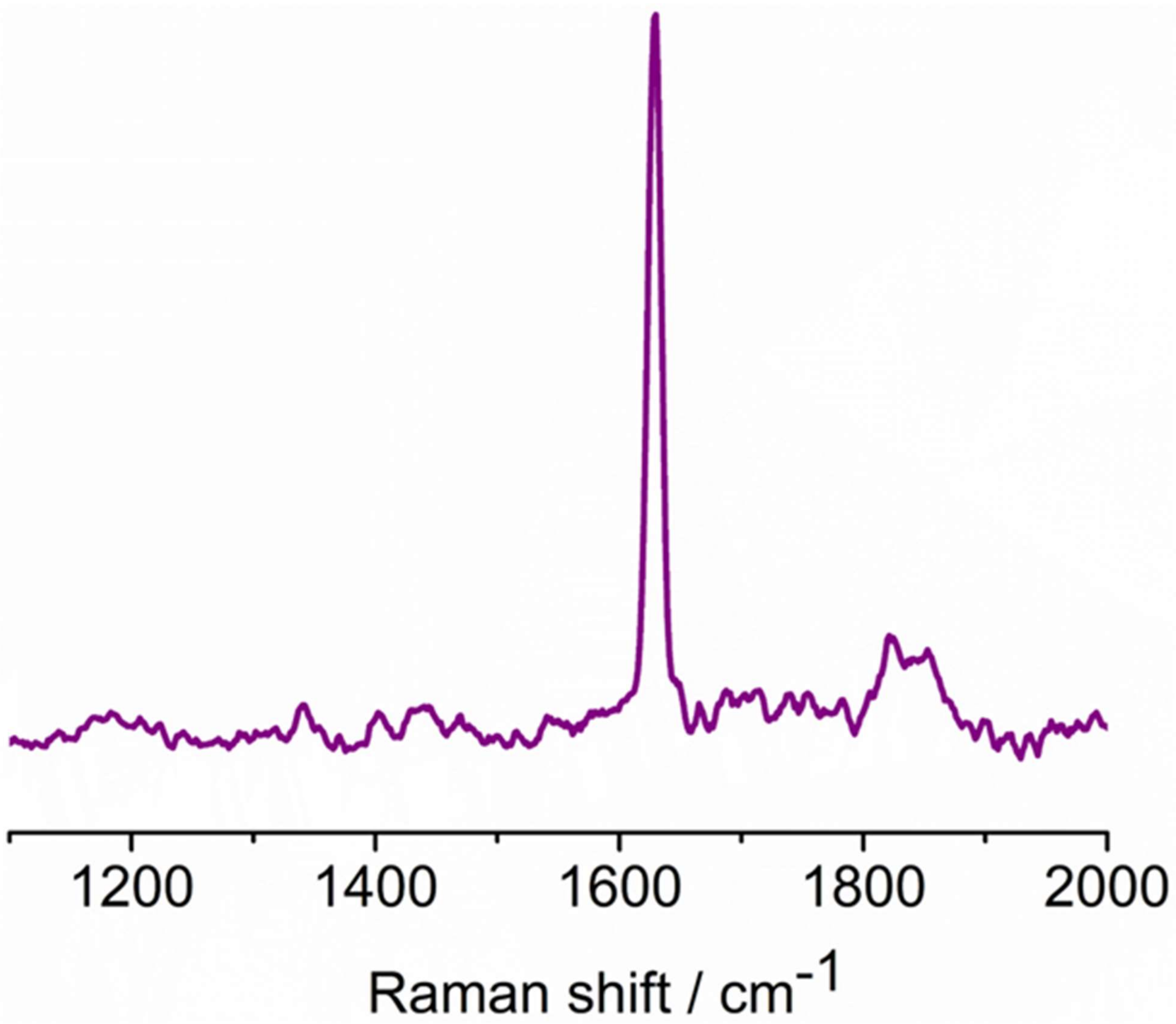

Figure S2. Raman spectrum of **1**$_Y$ ($\nu_{N=N}$ = 1630 cm^{-1}). The spectrum is normalised and baseline corrected.

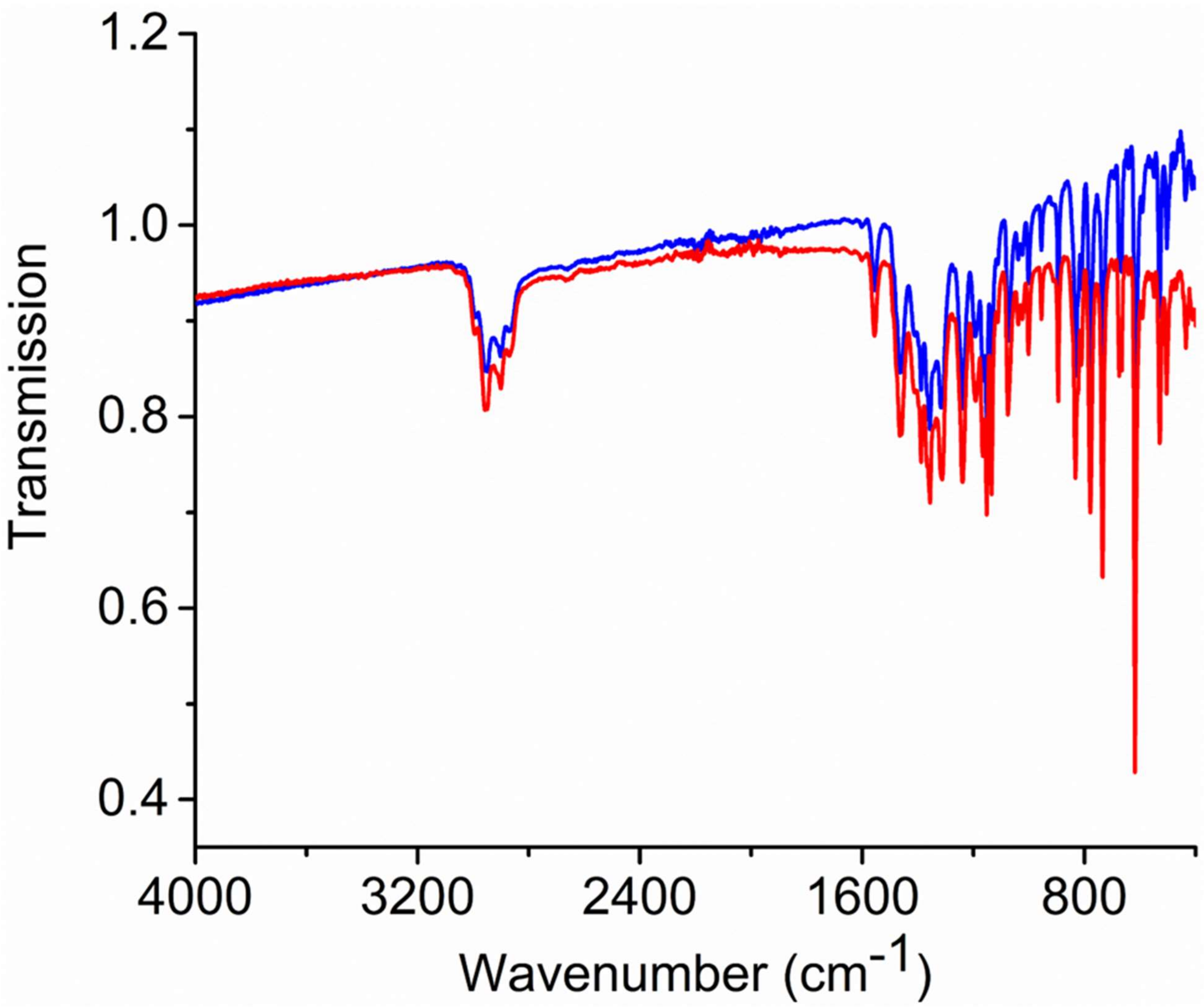

Figure S3. FTIR spectra of $\mathbf{2}_{\mathbf{Gd}}$ (blue) and $\mathbf{2}_{\mathbf{Tb}}$ (red).

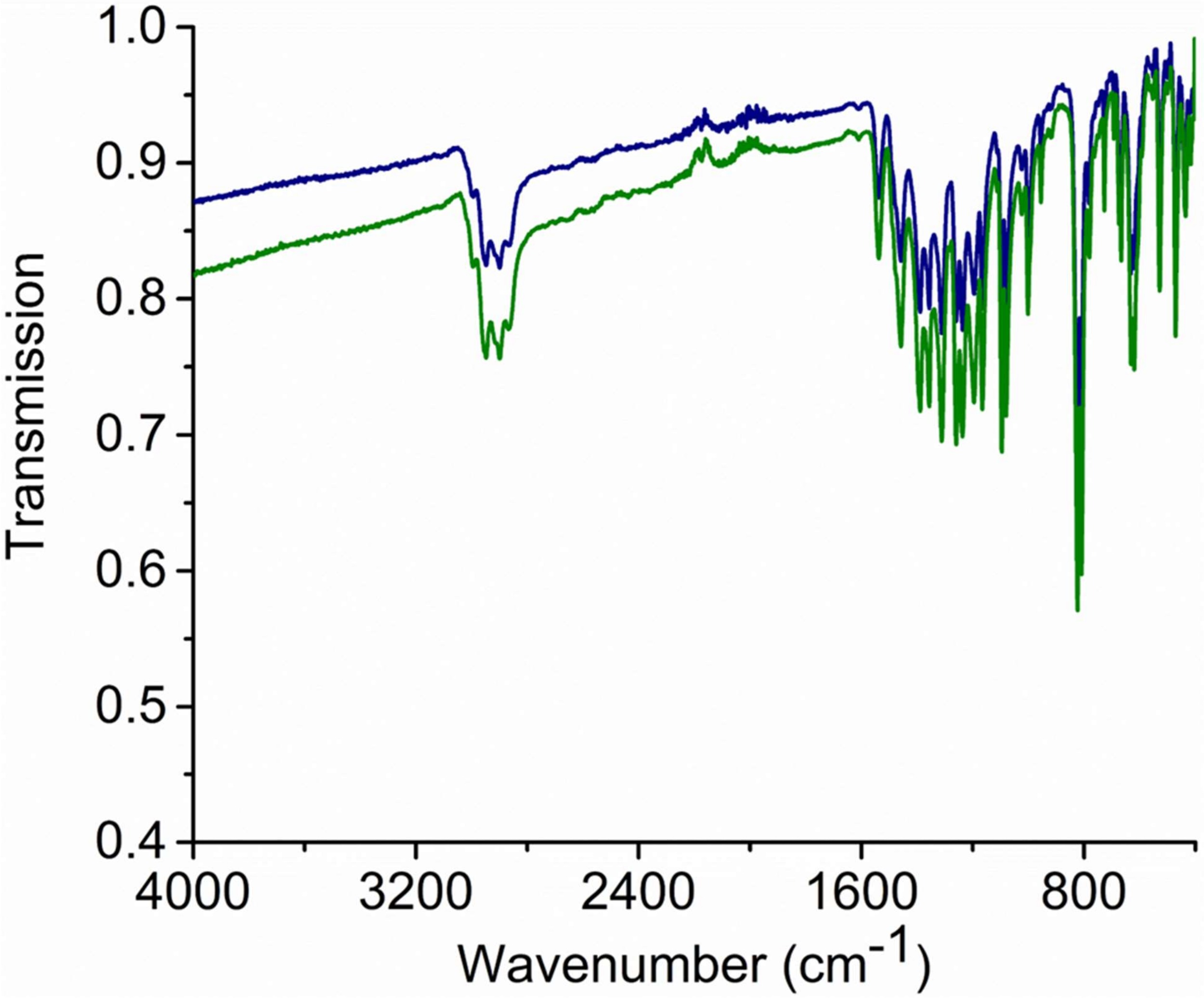

Figure S4. FTIR spectra of $\mathbf{3_Y}$ (blue) and $\mathbf{3_{Gd}}$ (green).

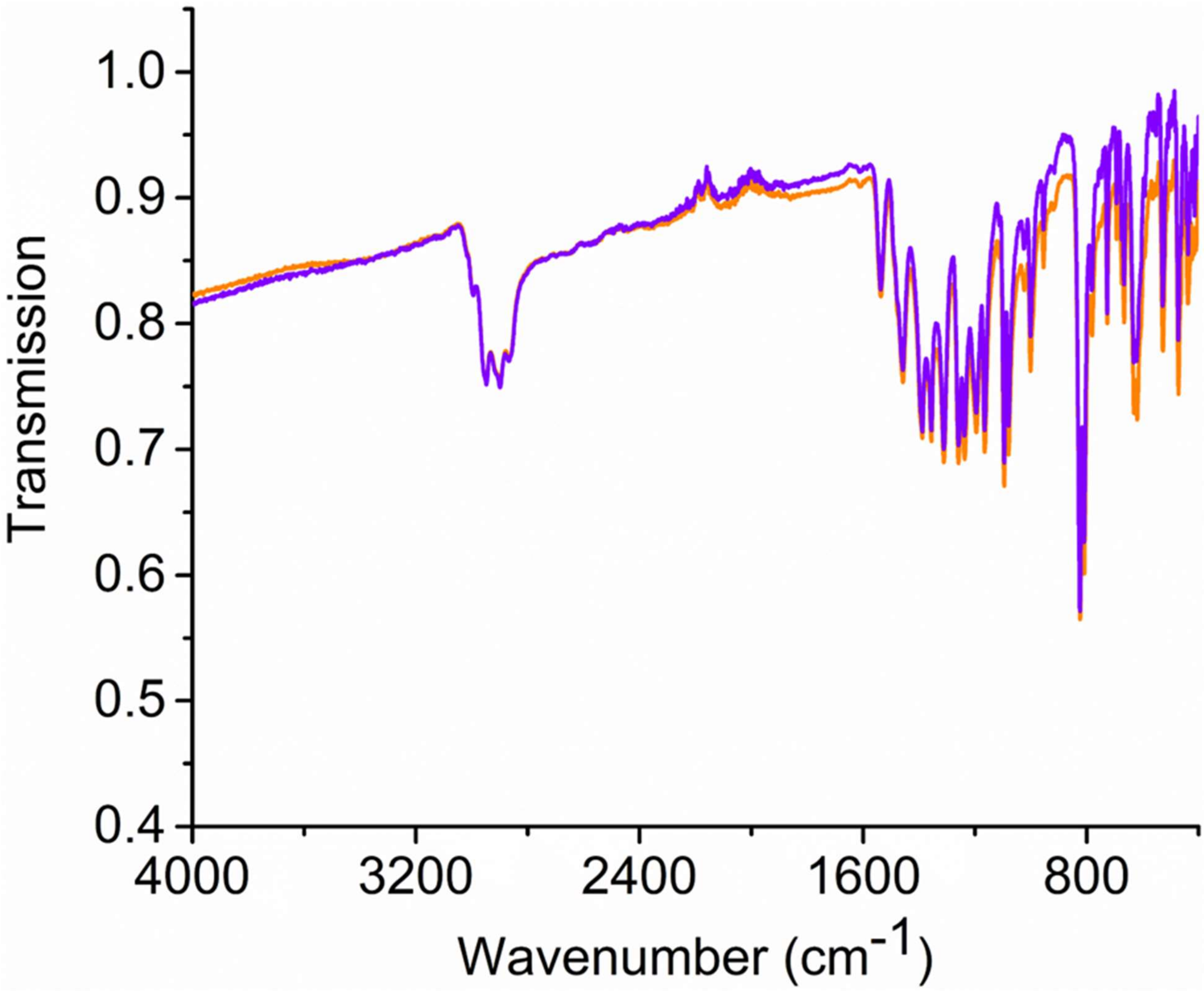

Figure S5. FTIR spectra of $\mathbf{3}_{\mathbf{Tb}}$ (orange) and $\mathbf{3}_{\mathbf{Dy}}$ (purple).

X-Ray Crystallography

Single-crystal X-ray diffraction measurements for complexes **1**$_{\mathbf{Y}}$, and **2**$_{\mathbf{Tb}}$, were carried out on an Agilent Gemini Ultra or a Rigaku HyPix 6000HE diffractometer using Cu-K$_\alpha$ radiation (λ = 1.54184 Å) at 100 K. A Bruker D8 Venture Metaljet diffractometer using Ga-K$_\alpha$ radiation (λ = 1.34139 Å) was used for **2**$_{\mathbf{Gd}}$, **3**$_{\mathbf{Y}}$, **3**$_{\mathbf{Gd}}$, **3**$_{\mathbf{Tb}}$ and **3**$_{\mathbf{Dy}}$. Structures were solved in Olex2 with SHELXT using intrinsic phasing and were refined with SHELXL using least-squares minimisation.[6,7] Anisotropic thermal parameters were used for non-hydrogen atoms and isotropic parameters for hydrogen atoms. Hydrogen atoms on carbons were added geometrically and refined using a riding model. Solvent masking was used in the refinement for **2**$_{\mathbf{Gd}}$ (5 benzene), **2**$_{\mathbf{Tb}}$ (2.1 THF and 0.3 toluene), **3**$_{\mathbf{Y}}$ (2.2 toluene), **3**$_{\mathbf{Tb}}$ (4.5 toluene) and **3**$_{\mathbf{Dy}}$ (2.5 toluene).

Table S1. Crystal data and structure refinement parameters for **1**$_{\mathbf{Y}}$.

	**1**$_{\mathbf{Y}}$
CCDC ref. code	2421135
Empirical formula	$C_{34}H_{58}NY$
Formula weight	569.72
Crystal system	Orthorhombic
Space group	*Pccn*
a (Å)	11.01890(10)
b (Å)	28.0043(3)
c (Å)	20.7699(2)
α (°)	90
β (°)	90
γ (°)	90
V (Å^3)	6409.11(11)
Z	8
ρ_{calc} (g cm^{-3})	1.181
F (000)	2464.0
Reflections collected	149332
Independent reflections	5729
R_{int} (%)	0.0741
GOF on F^2	1.062
R_1^{a}	0.0377
wR_2^{b}	0.0935

^{a} $R_1[I>2\sigma(I)] = \sum||F_o|-|F_c||/\sum|F_o|$; ^{b} wR_2[all data] $= [\sum\{w(F_o^2-F_c^2)^2\}/\sum\{w(F_o^2)^2\}]^{1/2}$

Table S2. Selected bond lengths (Å) and angles (°) for $\mathbf{1_Y}$.

	$\mathbf{1_Y}$
Y–C	Y1-C1: 2.697(3)<br>Y1-C2: 2.670(3)<br>Y1-C3: 2.713(2)<br>Y1-C4: 2.699(3)<br>Y1-C5: 2.641(3)<br>Y1-C18: 2.721(3)<br>Y1-C19: 2.647(3)<br>Y1-C20: 2.694(3)<br>Y1-C21: 2.679(3)<br>Y1-C22: 2.732(2)
Y–Cp^{ttt}_{cent}	2.3950(12)<br>2.4070(12)
Y1–N1	2.258(2)
N1–N1A	1.199(4)
Y1···Y1A	5.7147(11)
Cp^{ttt}_{cent}–Y–Cp^{ttt}_{cent}	143.01(4)

Table S3. Crystal data and structure refinement parameters for $\mathbf{2_{Gd}}$ and $\mathbf{2_{Tb}}$.

	$\mathbf{2_{Gd}}$	$\mathbf{2_{Tb}}$
CCDC ref. code	2421136	2421134
Empirical formula	$C_{156}H_{216}Gd_3N_6$	$C_{126}H_{186}N_6Tb_3$
Formula weight	2643.96	2261.56
Crystal system	orthorhombic	trigonal
Space group	*Pbcn*	*R*3
a (Å)	17.9012(12)	19.1314(4)
b (Å)	31.863(2)	19.1314(4)
c (Å)	24.0680(17)	34.9804(7)
α (°)	90	90
β (°)	90	90
γ (°)	90	120
V (Å^3)	13728.3(16)	11087.9(5)
Z	4	3
ρ_{calc} (g cm^{-3})	1.279	1.016
F (000)	5537.0	3537.0
Reflections collected	169308	59283
Independent reflections	19784	8810
R_{int} (%)	0.0821	0.1035
GOF on F^2	1.061	0.979
R_1[a]	0.0322	0.0542
wR_2[b]	0.0871	0.1580

[a] $R_1[I>2\sigma(I)] = \sum||F_o|-|F_c||/\sum|F_o|$; [b] wR_2[all data] $= [\sum\{w(F_o^2-F_c^2)^2\}/\sum\{w(F_o^2)^2\}]^{1/2}$

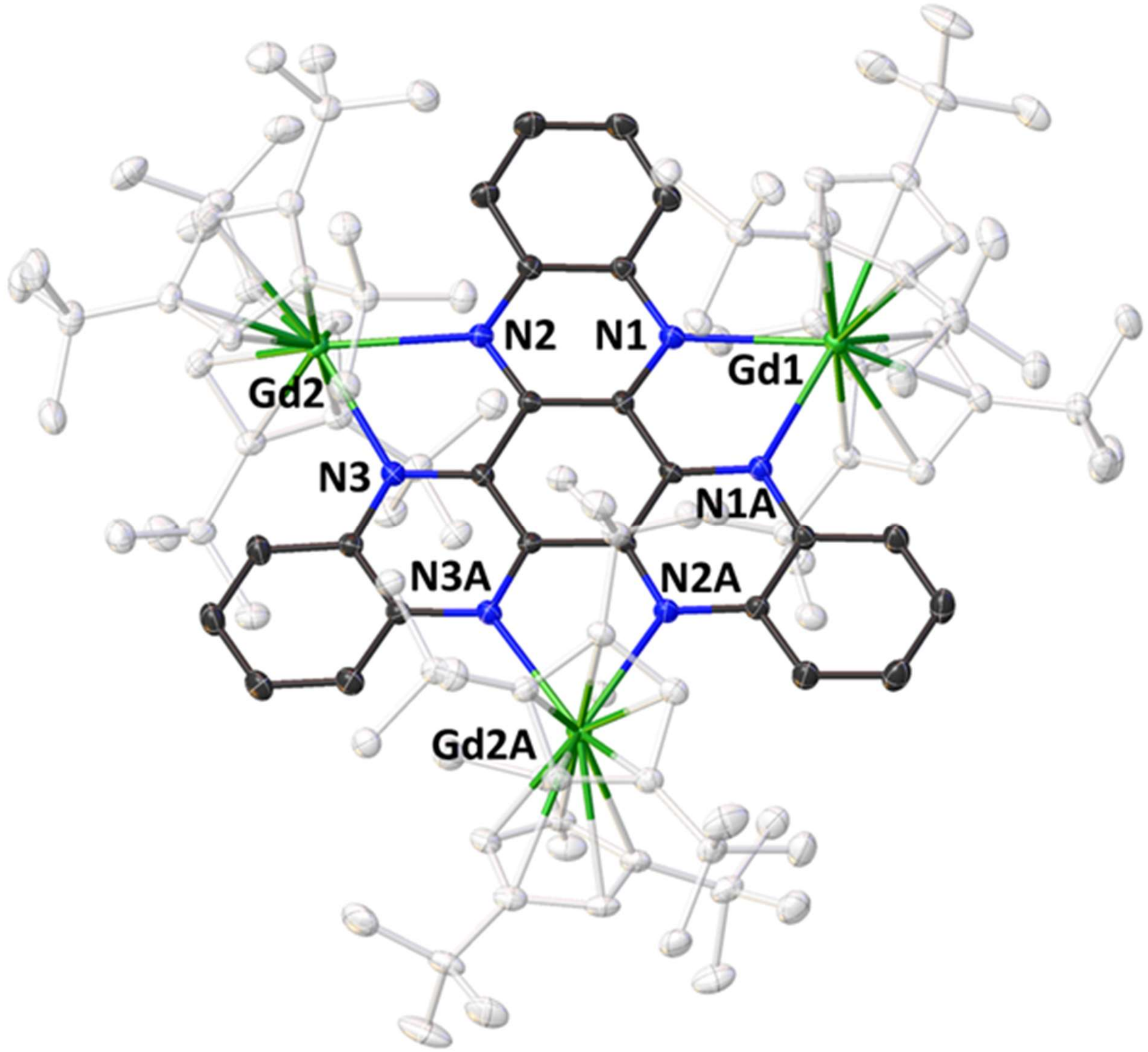

Figure S6. Molecular structure of $\mathbf{2}_{\mathbf{Gd}}$. Thermal ellipsoid representations set to 50% probability level and hydrogen atoms are omitted for clarity.

Table S4. Selected bond lengths (Å) and angles (°) for $\mathbf{2_{Gd}}$ and $\mathbf{2_{Tb}}$.

	$\mathbf{2_{Gd}}$	$\mathbf{2_{Tb}}$
M-C	Gd1-C1: 2.815(2)<br>Gd1-C2: 2.754(2)<br>Gd1-C3: 2.788(2)<br>Gd1-C4: 2.732(2)<br>Gd1-C5: 2.800(2)<br>Gd2-C18: 2.867(2)<br>Gd2-C19: 2.846(2)<br>Gd2-C20: 2.742(2)<br>Gd2-C21: 2.774(2)<br>Gd2-C22: 2.760(2)<br>Gd2-C35: 2.829(2)<br>Gd2-C36: 2.830(2)<br>Gd2-C37: 2.758(2)<br>Gd2-C38: 2.792(2)<br>Gd2-C51: 2.744(2)	Tb1-C1: 2.735(17)<br>Tb1-C2: 2.797(14)<br>Tb1-C3: 2.740(13)<br>Tb1-C4: 2.816(14)<br>Tb1-C5: 2.808(18)<br>Tb1-C18: 2.703(15)<br>Tb1-C19: 2.724(13)<br>Tb1-C20: 2.705(14)<br>Tb1-C21: 2.780(15)<br>Tb1-C22: 2.798(18)
M-Cp^{ttt}_{cent}	2.50058(13),<br>2.52289(16),<br>2.5146(2),	2.520(7)<br>2.484(7)
M-N	2.4899(17), 2.4844(19),<br>2.4809(17)	2.471(8)<br>2.480(10)
Cp^{ttt}_{cent}-M-Cp^{ttt}_{cent}	131.990(5), 130.9959(4)	131.1(2)

Table S5. Crystal data and structure refinement parameters for **3_Y**, **3_{Gd}**, **3_{Tb}** and **3_{Dy}**.

	**3_Y**	**3_{Gd}**	**3_{Tb}**	**3_{Dy}**
CCDC ref. code	2421137	2421139	2421140	2421138
Empirical formula	$C_{147.4}H_{215.6}N_6Y_3$	$C_{149.5}H_{218}Gd_3N_6$	$C_{163.5}H_{234}N_6Tb_3$	$C_{149.5}H_{218}Dy_3N_6$
Formula weight	2338.38	2571.04	2760.31	2586.79
Crystal system	monoclinic	monoclinic	monoclinic	monoclinic
Space group	$P2_1/n$	$P2_1/n$	$P2_1/n$	$P2_1/n$
a (Å)	17.8532(8)	17.6683(8)	17.9001(5)	17.7600(5)
b (Å)	24.5914(10)	24.8953(12)	24.5983(6)	24.7029(7)
c (Å)	32.1003(14)	31.8281(15)	32.0896(8)	31.9240(8)
α (°)	90	90	90	90
β (°)	90.332(2)	90.021(2)	90.2410(10)	90.0620(10)
γ (°)	90	90	90	90
V (Å^3)	14092.9(11)	13999.8(11)	14129.3(6)	14005.8(7)
Z	4	4	4	4
ρ_{calc} (g cm^{-3})	1.102	1.220	1.298	1.227
F (000)	5036.0	5396.0	5808.0	5420.0
Reflections collected	360559	480262	806780	399639
Independent reflections	25568	25788	26007	25658
R_{int} (%)	0.0563	0.0981	0.0844	0.1274
GOF on F^2	1.058	1.092	1.053	1.063
R_1[a]	0.0301	0.0768	0.0377	0.0579
wR_2[b]	0.0845	0.2276	0.1004	0.1695

[a] $R_1[I>2\sigma(I)] = \sum||F_o|-|F_c||/\sum|F_o|$; [b] wR_2[all data] $= [\sum\{w(F_o^2-F_c^2)^2\}/\sum\{w(F_o^2)^2\}]^{1/2}$

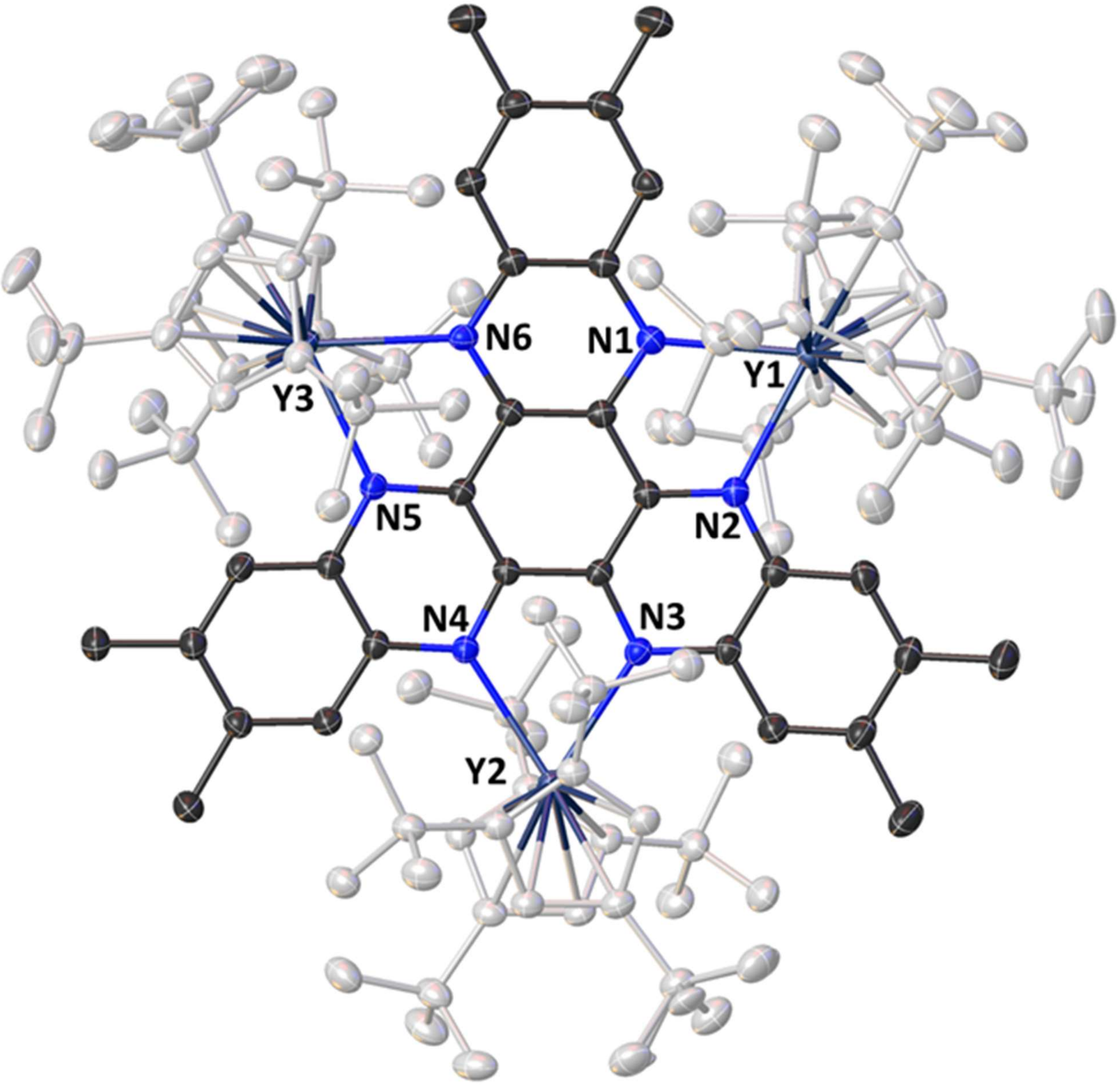

Figure S7. Molecular structure of $\mathbf{3_Y}$. Thermal ellipsoid representations set to 50% probability level and hydrogen atoms are omitted for clarity.

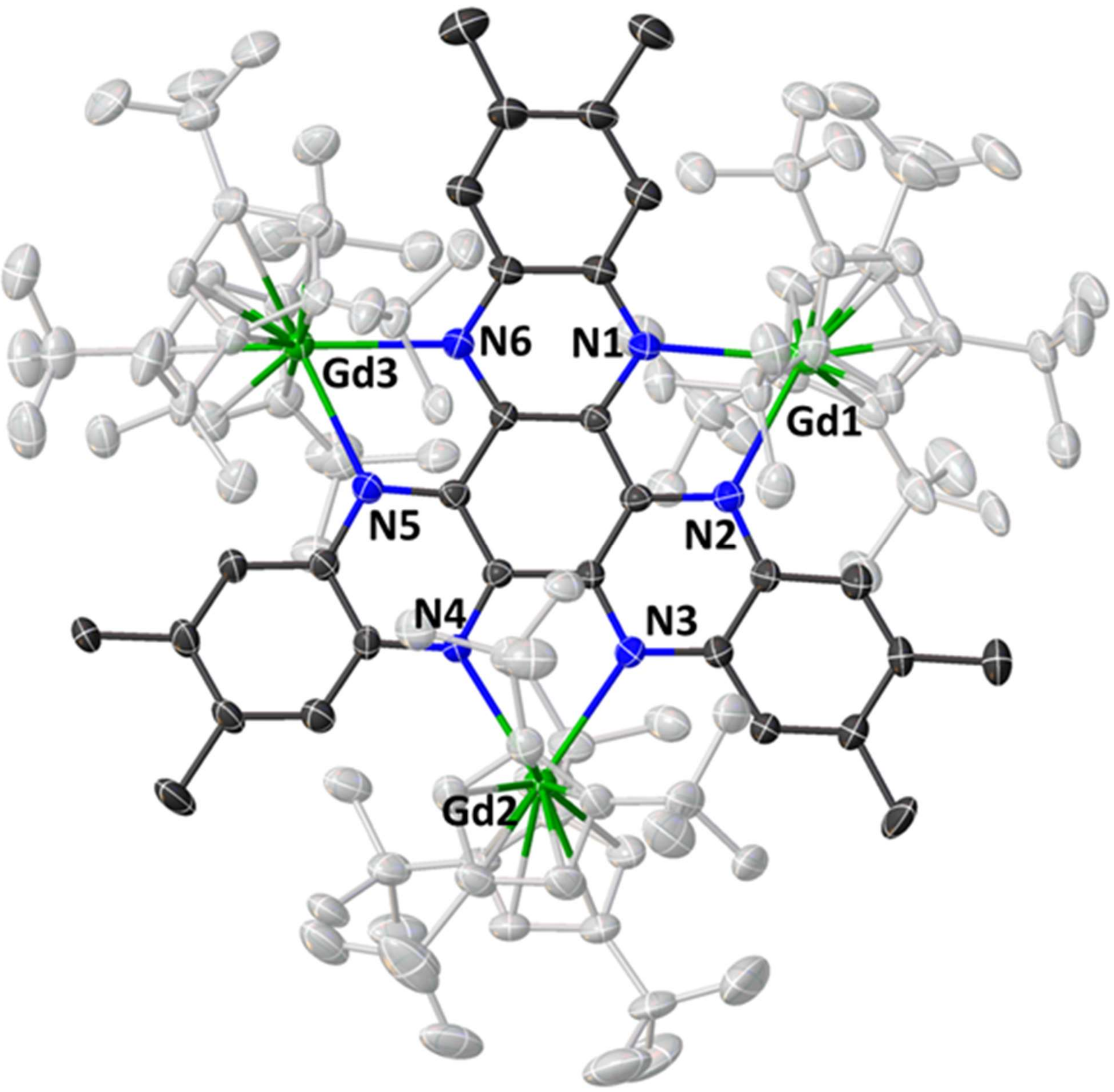

Figure S8. Molecular structure of $\mathbf{3_{Gd}}$. Thermal ellipsoid representations set to 50% probability level and hydrogen atoms are omitted for clarity.

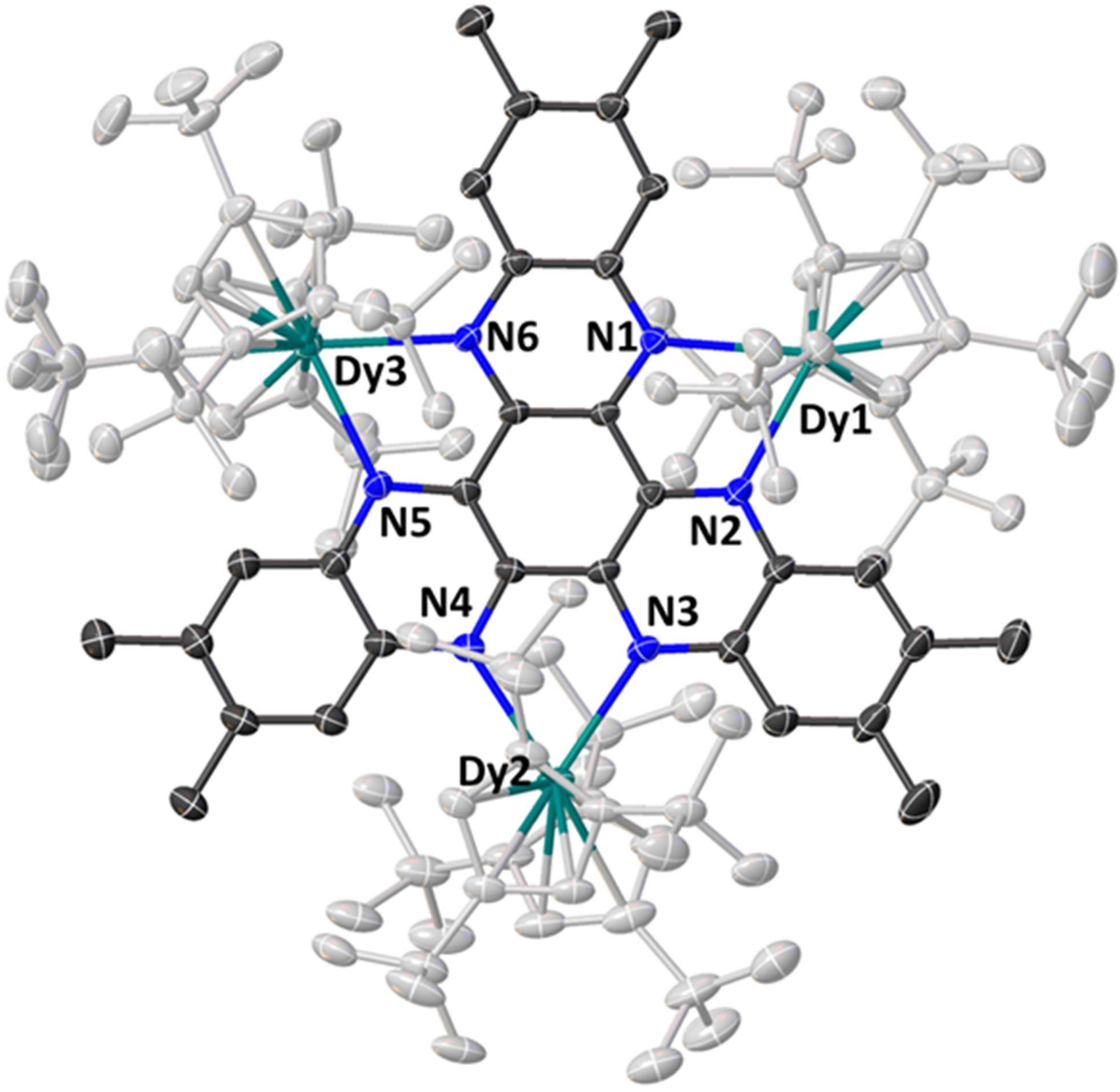

Figure S9. Molecular structure of $\mathbf{3_{Dy}}$. Thermal ellipsoid representations set to 50% probability level and hydrogen atoms are omitted for clarity.

Table S6. Selected bond lengths (Å) and angles (°) for $\mathbf{3}_{\mathbf{M}}$.

	$\mathbf{3}_{\mathbf{Y}}$	$\mathbf{3}_{\mathbf{Gd}}$	$\mathbf{3}_{\mathbf{Tb}}$	$\mathbf{3}_{\mathbf{Dy}}$
M-C	Y1-C1: 2.719(2)	Gd1-C41: 2.833(10)	Tb1-C1: 2.733(4)	Dy1-C1: 2.844(5)
	Y1-C2: 2.7989(19)	Gd1-C42: 2.859(11)	Tb1-C2: 2.774(4)	Dy1-C2: 2.831(6)
	Y1-C3: 2.7919(18)	Gd1-C43: 2.781(11)	Tb1-C3: 2.737(4)	Dy1-C3: 2.720(7)
	Y1-C4: 2.707(2)	Gd1-C44: 2.799(11)	Tb1-C4: 2.817(4)	Dy1-C4: 2.768(6)
	Y1-C5: 2.758(2)	Gd1-C45: 2.764(10)	Tb1-C5: 2.802(4)	Dy1-C5: 2.733(5)
	Y1-C18: 2.756(2)	Gd1-C51: 2.815(12)	Tb1-C18: 2.721(4)	Dy1-C18: 2.788(7)
	Y1-C19: 2.828(2)	Gd1-C52: 2.854(14)	Tb1-C19: 2.791(4)	Dy1-C19: 2.794(5)
	Y1-C20: 2.7840(18)	Gd1-C53: 2.790(14)	Tb1-C20: 2.840(4)	Dy1-C20: 2.754(6)
	Y1-21: 2.7080(18)	Gd1-C54: 2.779(14)	Tb1-C21: 2.765(4)	Dy1-C21: 2.811(7)
	Y1-22: 2.781(2)	Gd1-C55: 2.729(12)	Tb1-C22: 2.794(4)	Dy1-C22: 2.752(7)
	Y2-C35: 2.734(2)	Gd2-C61: 2.839(11)	Tb2-C35: 2.728(4)	Dy2-C35: 2.791(5)
	Y2-C36: 2.825(2)	Gd2-C62: 2.849(10)	Tb2-C36: 2.838(4)	Dy2-C36: 2.713(7)
	Y2-C37: 2.812(2)	Gd2-C63: 2.769(13)	Tb2-C37: 2.857(4)	Dy2-C37: 2.753(7)
	Y2-C38: 2.719(2)	Gd2-C64: 2.776(11)	Tb2-C38: 2.757(4)	Dy2-C38: 2.770(7)
	Y2-C39: 2.768(2)	Gd2-C65: 2.757(12)	Tb2-C39: 2.779(4)	Dy2-C39: 2.839(7)
	Y2-C92: 2.8486(18)	Gd2-C71: 2.827(12)	Tb2-C52: 2.739(4)	Dy2-C52: 2.713(7)
	Y2-C93: 2.751(2)	Gd2-C72: 2.841(12)	Tb2-C53: 2.816(4)	Dy2-C53: 2.753(7)
	Y2-C94: 2.764(2)	Gd2-C73: 2.740(12)	Tb2-C54: 2.836(4)	Dy2-C54: 2.740(7)
	Y2-C95: 2.7140(19)	Gd2-C74: 2.820(12)	Tb2-C55: 2.744(4)	Dy2-C55: 2.796(7)
	Y2-C96: 2.8242(18)	Gd2-C75: 2.746(13)	Tb2-C56: 2.794(4)	Dy2-C56: 2.806(7)
	Y3-C55: 2.7435(19)	Gd3-C81: 2.833(12)	Tb3-C72: 2.761(4)	Dy3-C69: 2.799(7)
	Y3-C56: 2.7907(18)	Gd3-C82: 2.799(12)	Tb3-C73: 2.747(4)	Dy3-C70: 2.720(7)
	Y3-C57: 2.7916(18)	Gd3-C83: 2.782(12)	Tb3-C74: 2.853(4)	Dy3-C71: 2.788(7)
	Y3-C58: 2.7443(18)	Gd3-C84: 2.859(12)	Tb3-C75: 2.854(4)	Dy3-C72: 2.721(7)
	Y3-C59: 2.819(2)	Gd3-C85: 2.786(12)	Tb3-C76: 2.749(4)	Dy3-C73: 2.813(7)
	Y3-C75: 2.7242(18)	Gd3-C91: 2.865(11)	Tb3-C89: 2.756(4)	Dy3-C89: 2.818(7)
	Y3-C76: 2.8426(18)	Gd3-C92: 2.908(10)	Tb3-C90: 2.811(4)	Dy3-C90: 2.716(7)
	Y3-C77: 2.8400(18)	Gd3-C93: 2.738(12)	Tb3-C91: 2.811(4)	Dy3-C91: 2.762(7)
	Y3-C78: 2.7285(19)	Gd3-C94: 2.769(11)	Tb3-C92: 2.762(4)	Dy3-C92: 2.744(7)
	Y3-C79: 2.7466(19)	Gd3-C95: 2.744(12)	Tb3-C93: 2.836(4)	Dy3-C93: 2.852(5)
M-Cp^{ttt}_{cent}	2.4763(10)	2.530(5)	2.4973(18)	2.505(3)
	2.4941(10)	2.524(6)	2.5054(18)	2.511(3)
	2.4945(10)	2.525(5)	2.5190(17)	2.486(3)
	2.5033(10)	2.518(6)	2.5207(16)	2.499(3)
	2.5004(9)	2.546(5)	2.5175(17)	2.494(3)
	2.5010(9)	2.533(5)	2.5105(17)	2.502(3)
M-N	2.4475(15)	2.466(9)	2.486(3)	2.474(4)
	2.4699(17)	2.471(9)	2.461(3)	2.447(4)
	2.4508(17)	2.491(7)	2.458(3)	2.455(5)
	2.4523(15)	2.482(7)	2.483(3)	2.475(5)
	2.4687(15)	2.490(7)	2.469(3)	2.456(4)
	2.4439(17)	2.482(9)	2.464(3)	2.470(4)
Cp^{ttt}_{cent}-M-Cp^{ttt}_{cent}	130.34(3)	130.20(17)	129.71(6)	130.63(10)
	129.31(4)	131.6(19)	130.66(6)	129.68(10)
	131.02(4)	131.13(18)	131.25(6)	130.09(1)

DFT Calculations on 1_Y

DFT calculations on $\mathbf{1_Y}$ were performed on the coordinates obtained from the X-ray structure using the ORCA 6.0.0 software package.[8] The position of hydrogen atoms was optimized at the DFT level using the pure GGA PBE exchange-correlation functional, keeping the positions of other atoms constant.[9,10] The CAM-B3LYP functional[11] was used and relativistic effects were included with the Zero-Order Regular Approximation (ZORA), together with relativistic segmented all-electron relativistically contracted (SARC)[12] version of the basis functions def2-TZVP for yttrium, nitrogen carbon and hydrogen.[13] The SARC/J auxiliary basis set and RIJCOSX approximation[14,15] with TightSCF convergence threshold were also used throughout. The TD-DFT calculations were performed with 50 excited states with a CPCM implicit solvent model for toluene.[16,17]

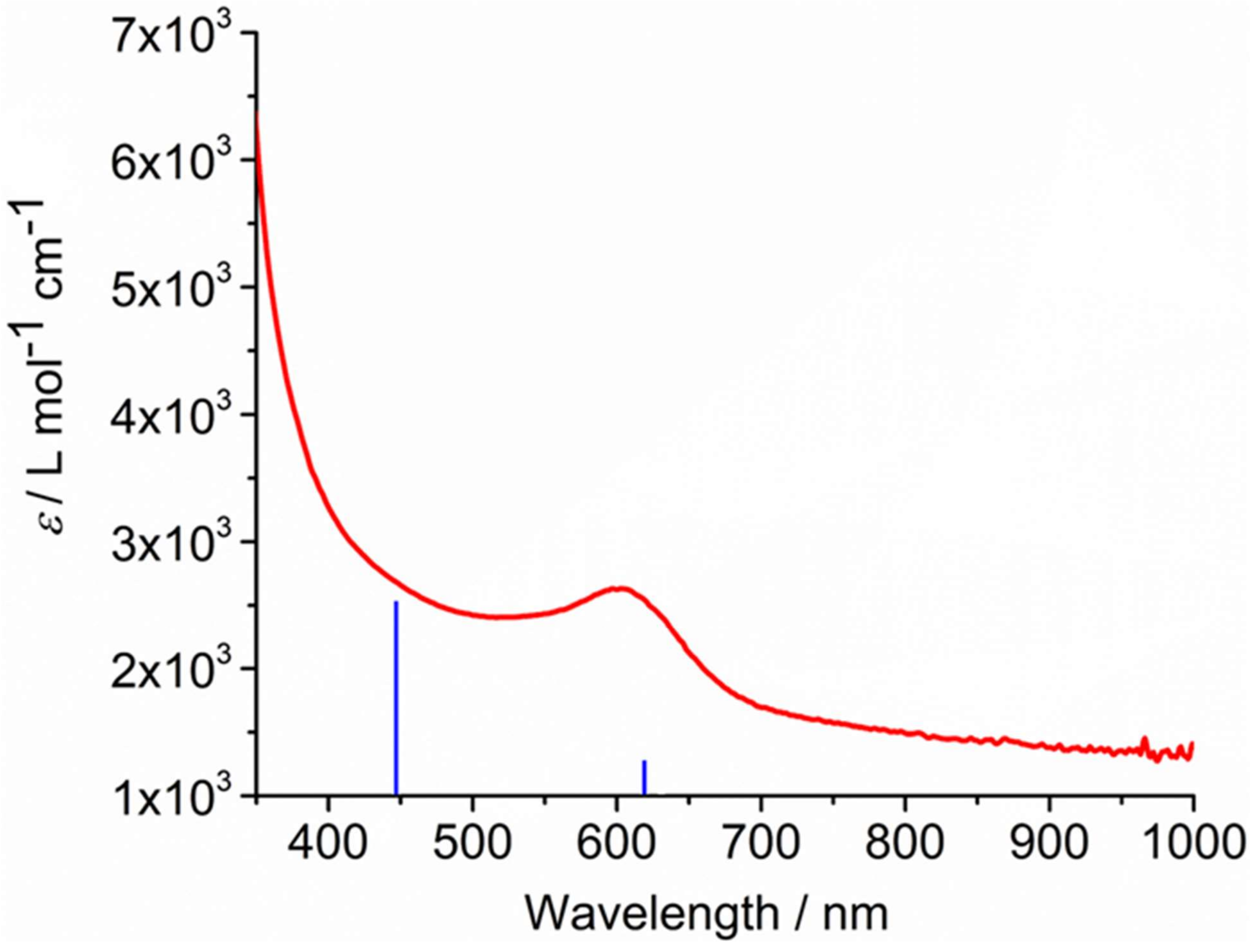

Figure S10. UV-vis spectrum in toluene for $\mathbf{1_Y}$ (λ_{max} = 603 nm). Solid vertical blue lines represent the TD-DFT calculated spectrum using the CAM-B3LYP/def-TZVP level of theory.

Table S7. Computed excitation wavelengths (λ) and oscillator strengths (*f*) in length representation for $\mathbf{1}_{\mathbf{Y}}$.

Excitation	λ / nm	*f*	Assignment (major contribution)
307a → 309a	630.0	0.00012548	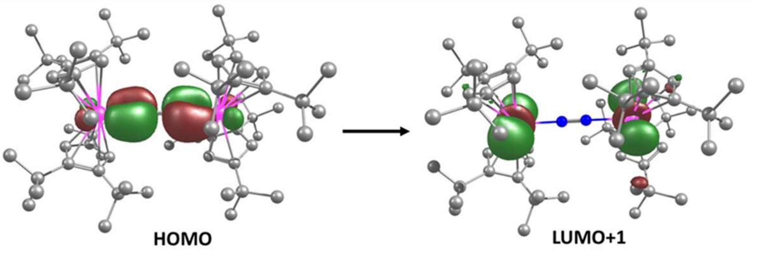
307a → 310a	618	0.07281	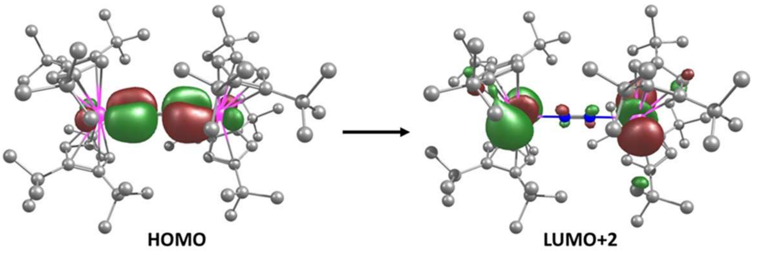
307a → 311a	446	0.40703	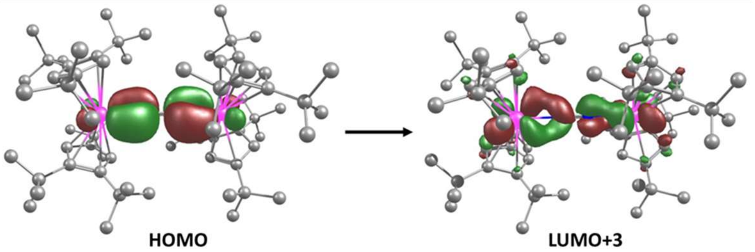

EPR Spectroscopy

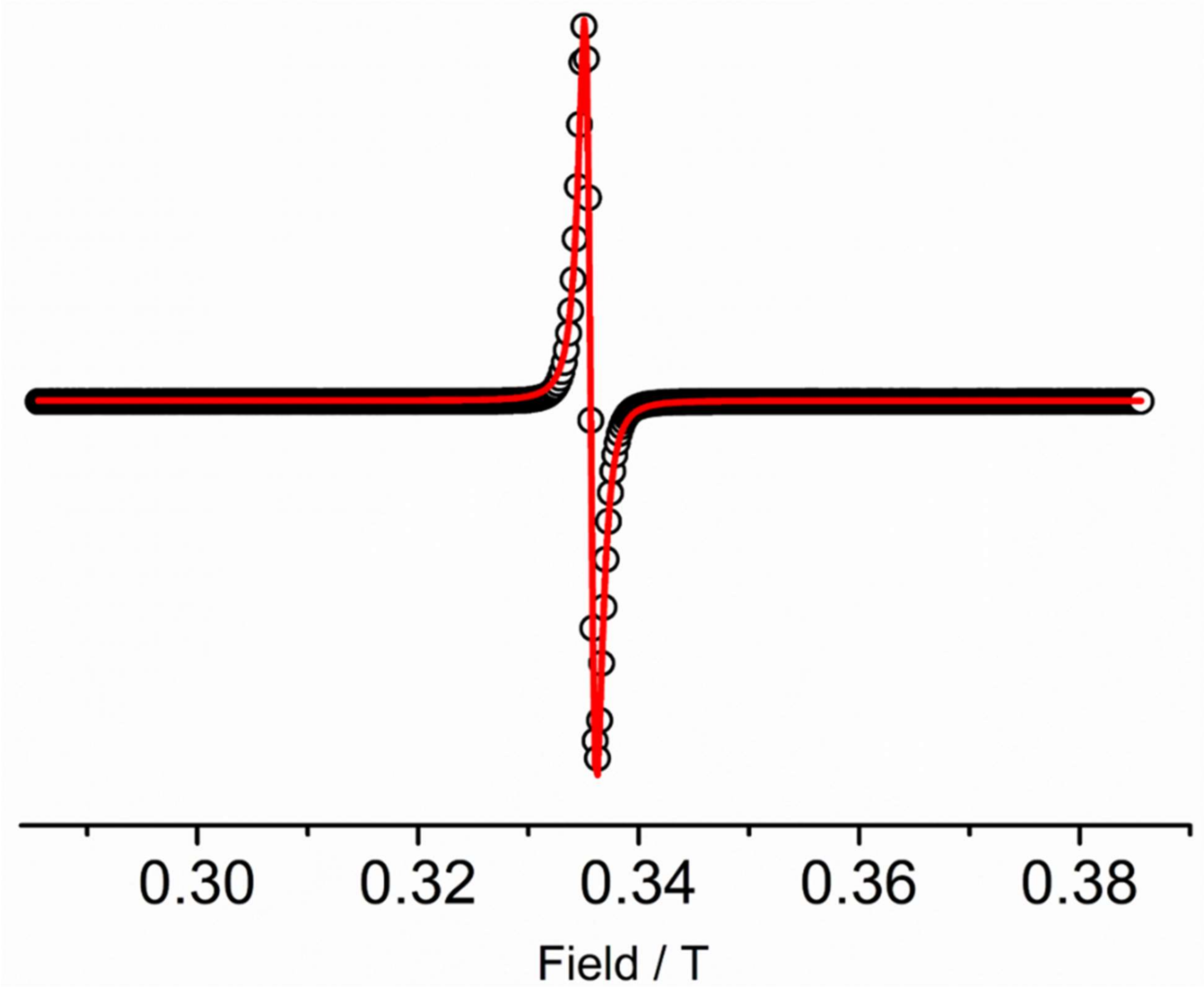

Figure S11. X-band EPR spectrum for **3**$_Y$ in the solid state at 298 K. The red line is a fit of the spectrum (*g* = 2.009 ± 0.0000052, lwpp = 0.049 ± 0.00008).

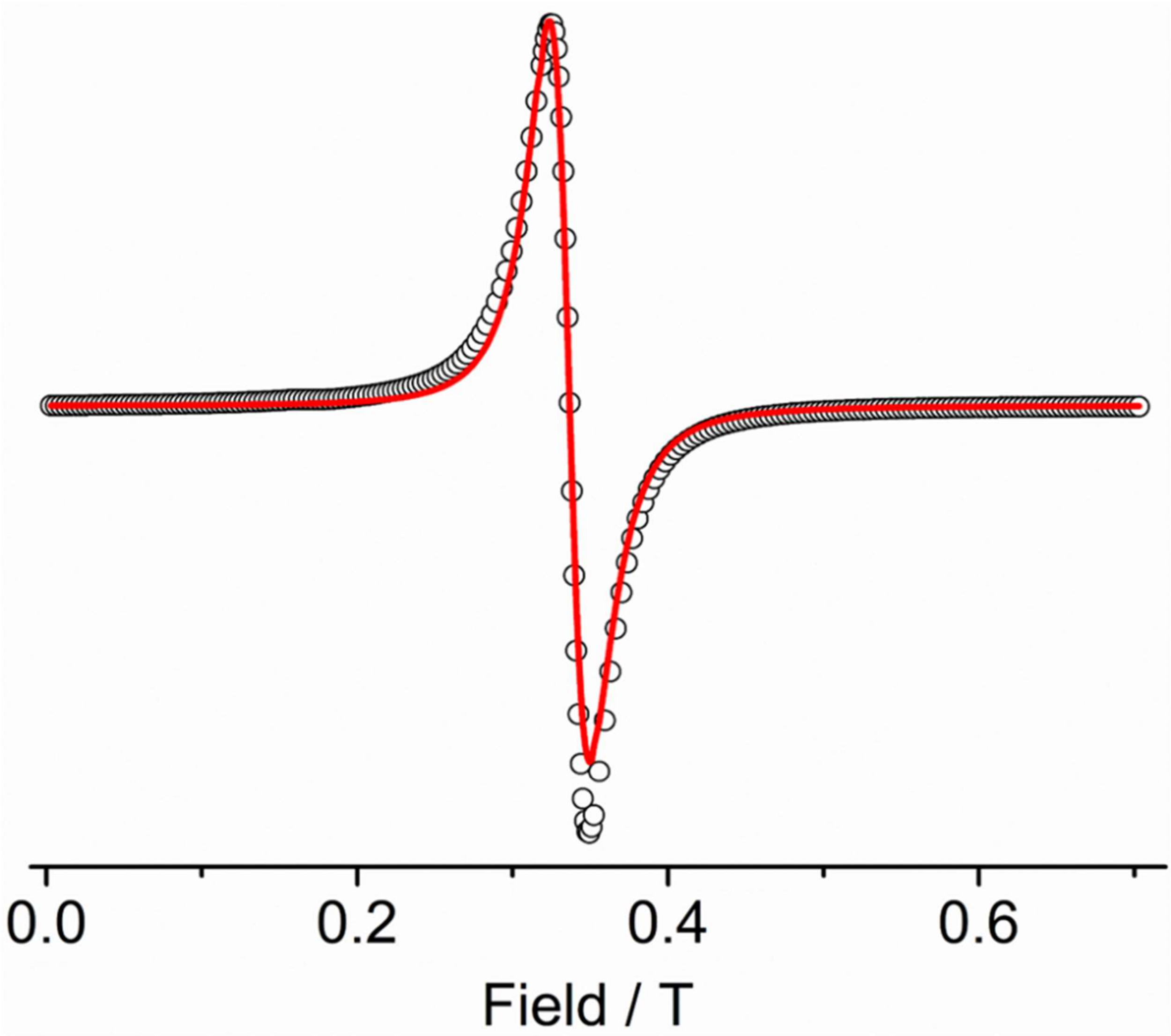

Figure S12. X-band EPR spectrum for $\mathbf{2}_{Gd}$ in the solid state at 298 K. The red line is a fit of the spectrum (g = 2.007 ± 0.0002, lwpp = 1.271 ± 0.0039).

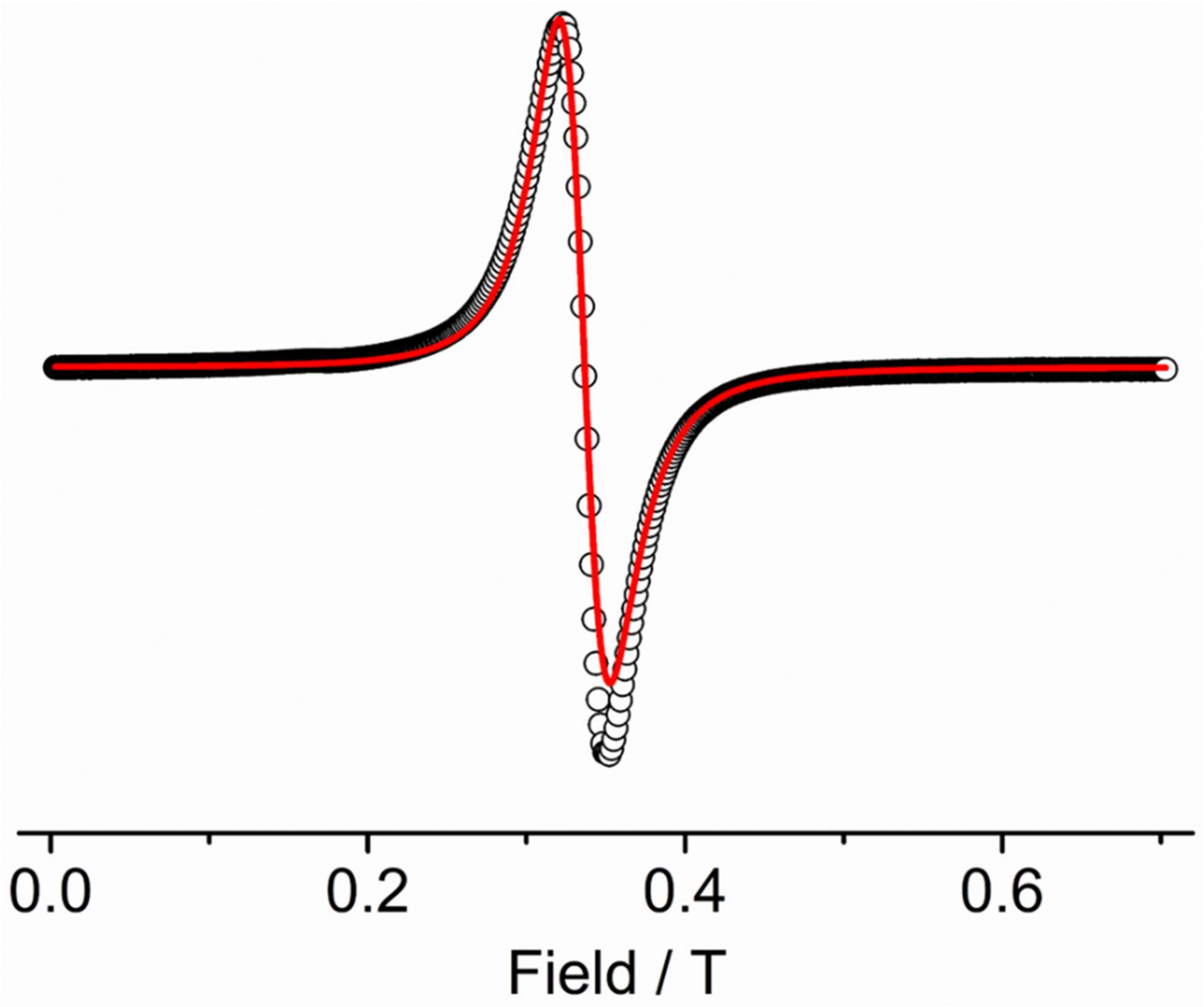

Figure S13. X-band EPR spectrum for $\mathbf{3_{Gd}}$ in the solid state at 298 K. The red line is a fit of the spectrum (g = 2.009 ± 0.0004, lwpp = 1.558 ± 0.0083).

Magnetic Property Measurements

Magnetic measurements were performed using a Quantum Design MPMS3 SQUID magnetometer equipped with a 7 T magnet. Samples were prepared by gently crushing the crystalline materials before transferring them to a 7 mm NMR tube and covering them in eicosane. Then the tubes were flame sealed under a static vacuum. The eicosane was melted in a water bath at 40 °C to prevent crystallite torquing. An applied field of 1000 Oe was used for all susceptibility measurements. Diamagnetic corrections were performed using Pascal's constants.[18]

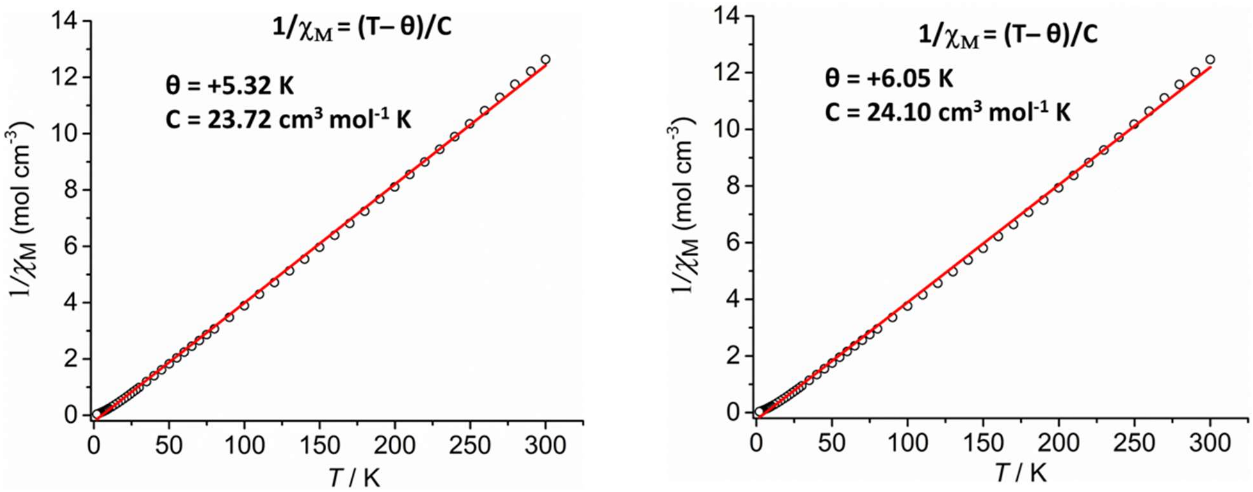

Figure S14. $1/\chi_{\mathrm{M}}(T)$ data for $\mathbf{2_{Gd}}$ (left) and $\mathbf{3_{Gd}}$ (right). The fits (red lines) produce positive Weiss constants, θ, indicating ferromagnetic exchange between the metal centres and the radical ligand.

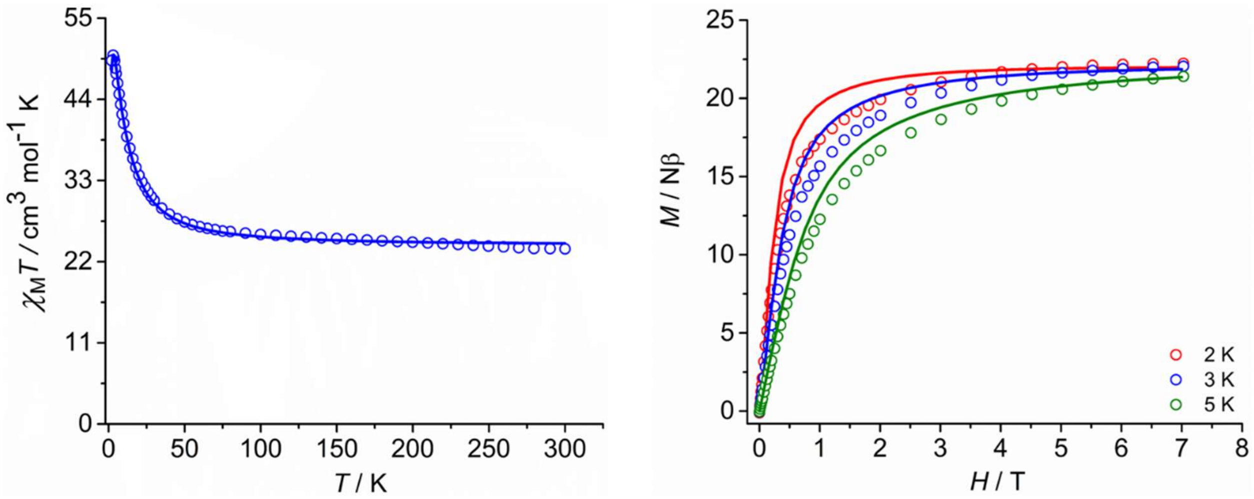

Figure S15. Left: $\chi_{\mathrm{M}}T(T)$ for $\mathbf{2_{Gd}}$. $\chi_{\mathrm{M}}T$ is 23.82 cm^3 K mol^{-1} at 300 K and 49.23 cm^3 K mol^{-1} at 2 K. Right: $M(H)$ data at 2, 3 and 5 K. The magnetizations is 22.17 Nβ at 7 T and 2 K. The solid lines are fits of the data according to equation 1, below.

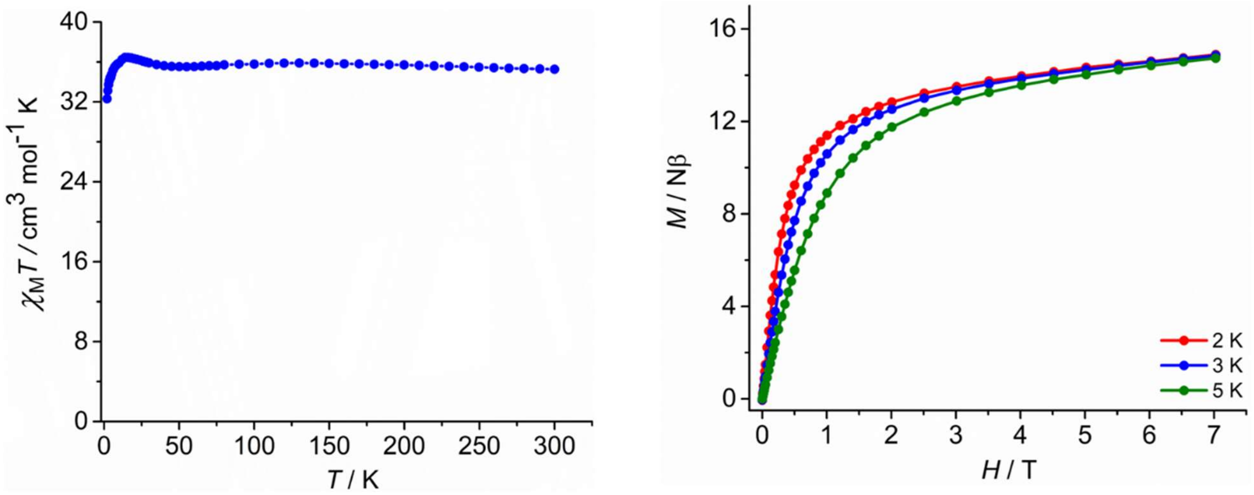

Figure S16. Left: $\chi_{\mathrm{M}}T(T)$ for $\mathbf{2_{Tb}}$. The $\chi_{\mathrm{M}}T$ values are 35.26, 36.46 and 32.31 cm^3 K mol^{-1} at 300, 14 and 2 K temperatures respectively. Right: $M(H)$ data at 2, 3 and 5 K. The magnetization is 14.88 Nβ at 7 T and 2 K.

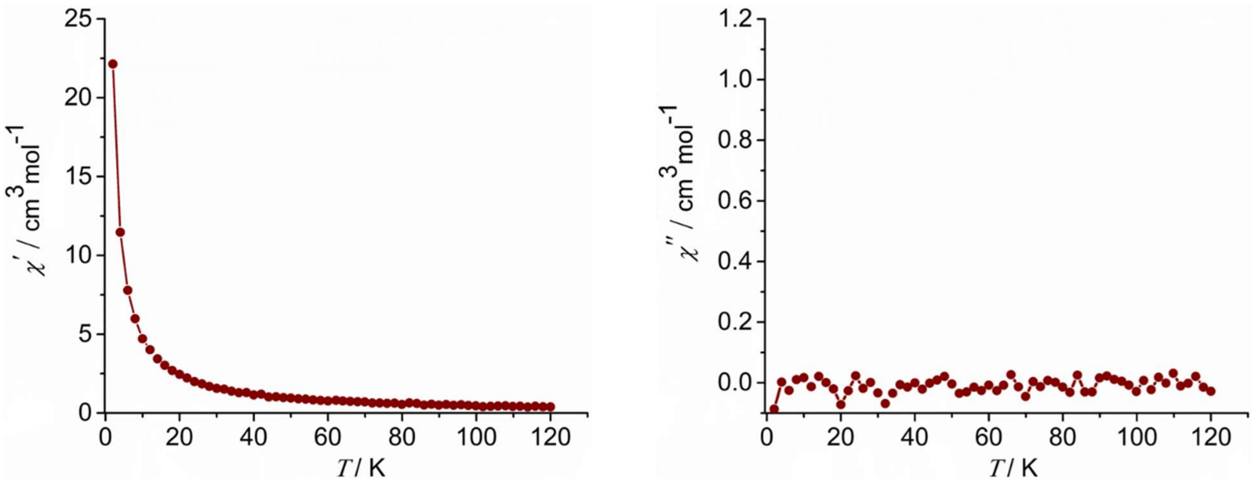

Figure S17. Real (left) and imaginary (right) components of the AC susceptibility as a function of temperature at 1000 Hz in an AC field of 3 Oe and zero DC field for $\mathbf{2_{Tb}}$.

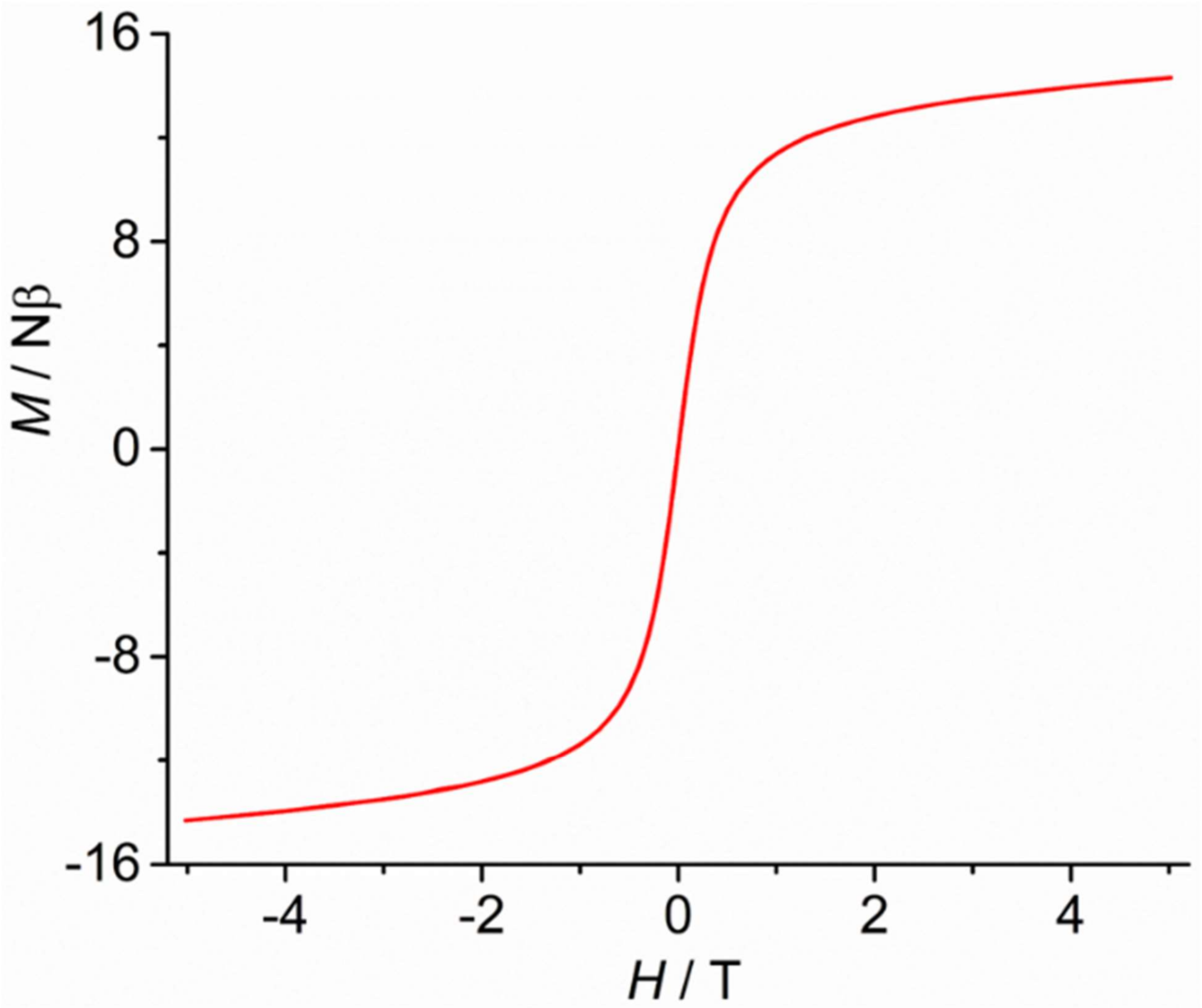

Figure S18. Magnetic hysteresis plot for $\mathbf{2}_{\mathbf{Tb}}$. The data were continuously collected at 1.9 K under the following field sweep rates: 5 mT s^{-1} |0-0.02| T; 10 mT s^{-1} |0.02-0.1| T; 50 mT s^{-1} |0.1-0.40| T; 100 mT s^{-1} |0.4-2.0| T; 200 mT s^{-1} |2.0-3.0| T; 500 mT s^{-1} |3.0-5.0| T.

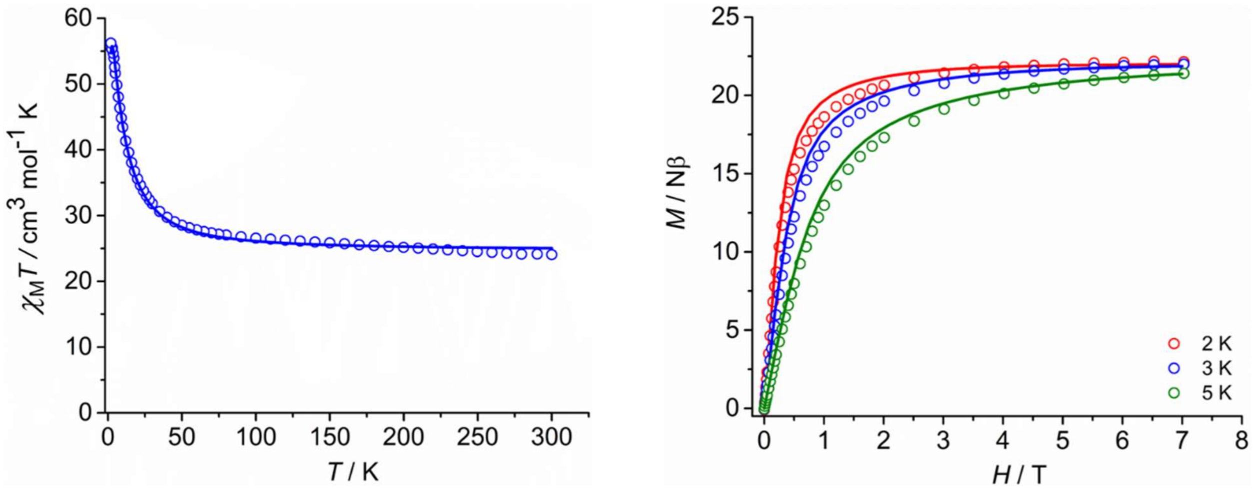

Figure S19. Left: $\chi_M T(T)$ for $\mathbf{3}_{Gd}$. $\chi_M T$ is 24.06 cm^3 K mol^{-1} at 300 K and 56.22 cm^3 K mol^{-1} at 2 K. Right: $M(H)$ data at 2, 3 and 5 K. Magnetizations value reaches 22.14 Nβ at 7 T and 2 K temperature. The solid lines represent a fit of the data according to Equation 1, below.

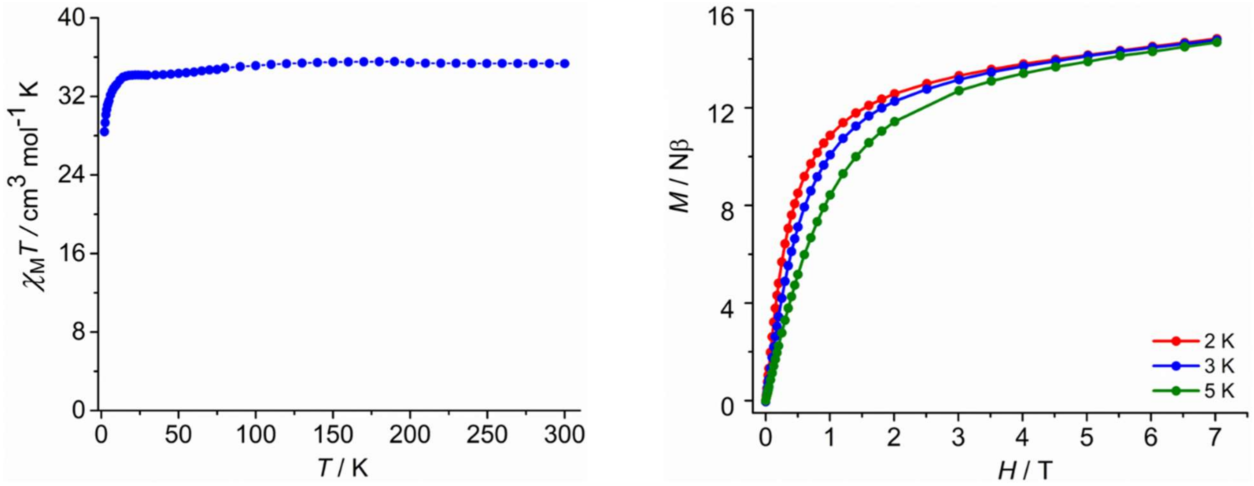

Figure S20. Left: $\chi_M T(T)$ for $\mathbf{3}_{Tb}$. The $\chi_M T$ values are 35.34 and 28.40 cm^3 K mol^{-1} at 300 and 2 K, respectively. Right: $M(H)$ data at 2, 3 and 5 K. The magnetization is 14.82 Nβ at 7 T and 2 K.

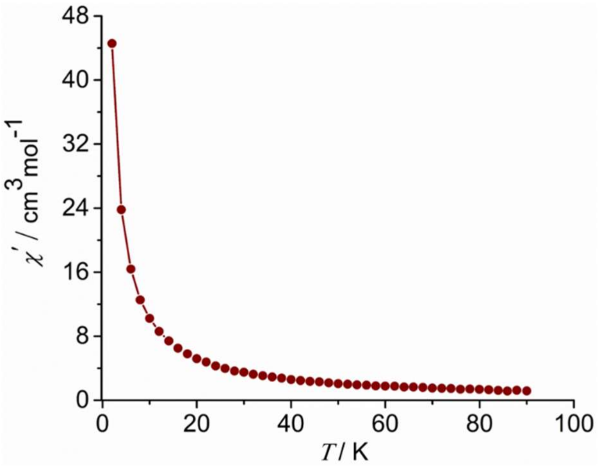

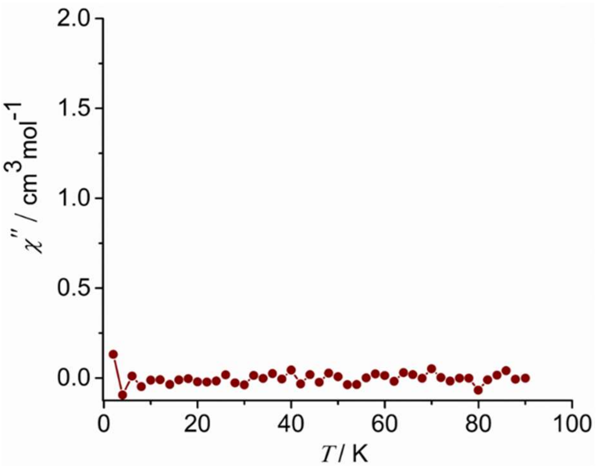

Figure S21. Real (left) and imaginary (right) components of the AC susceptibility as a function of temperature at 1000 Hz in an AC field of 3 Oe and zero DC field for $\mathbf{3_{Tb}}$.

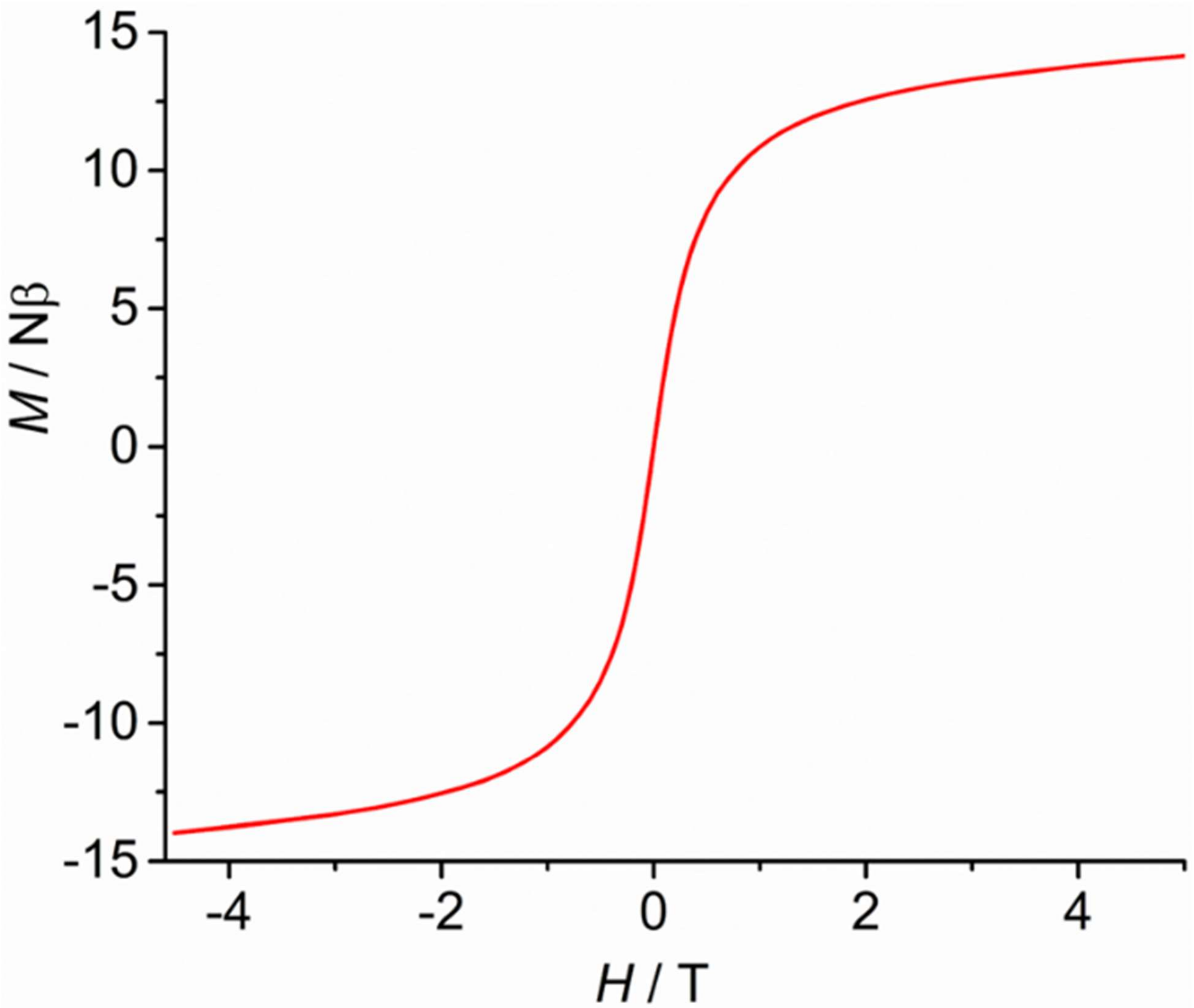

Figure S22. Magnetic hysteresis plot for $\mathbf{3}_{Tb}$. The data were continuously collected at 1.9 K under the following field sweep rates: 5 mT s^{-1} |0-0.02| T; 10 mT s^{-1} |0.02-0.1| T; 50 mT s^{-1} |0.1-0.40| T; 100 mT s^{-1} |0.4-2.0| T; 200 mT s^{-1} |2.0-3.0| T; 500 mT s^{-1} |3.0-5.0| T.

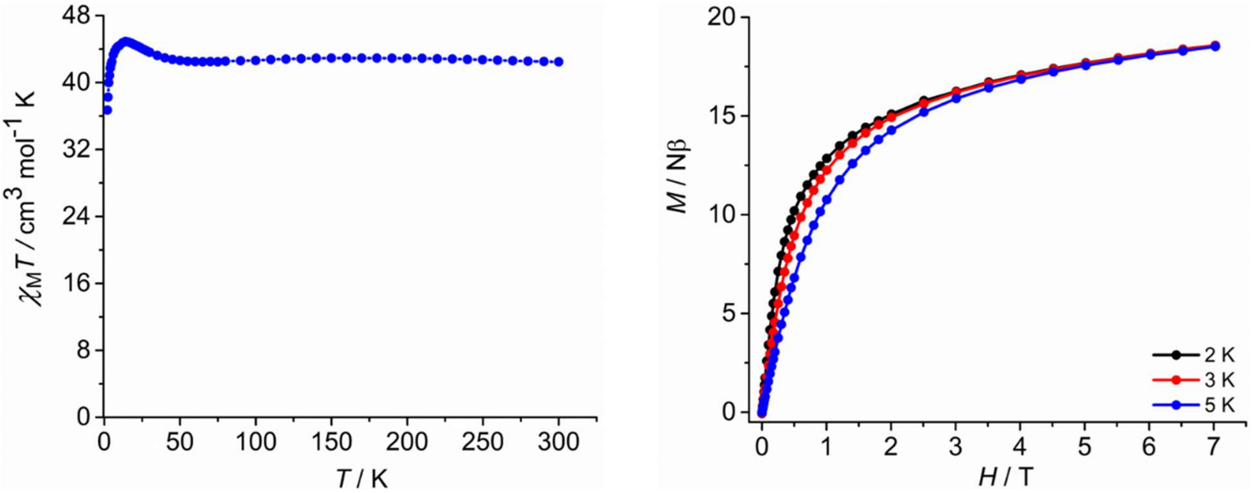

Figure S23. Left: $\chi_M T(T)$ for $\mathbf{3_{Dy}}$. The $\chi_M T$ values are 42.48, 44.49 and 36.71 cm^3 K mol^{-1} at 300, 14 and 2 K temperatures respectively. Right: $M(H)$ data at 2, 3 and 5 K. Magnetizations value reaches 14.88 Nβ at 7 T and 2 K.

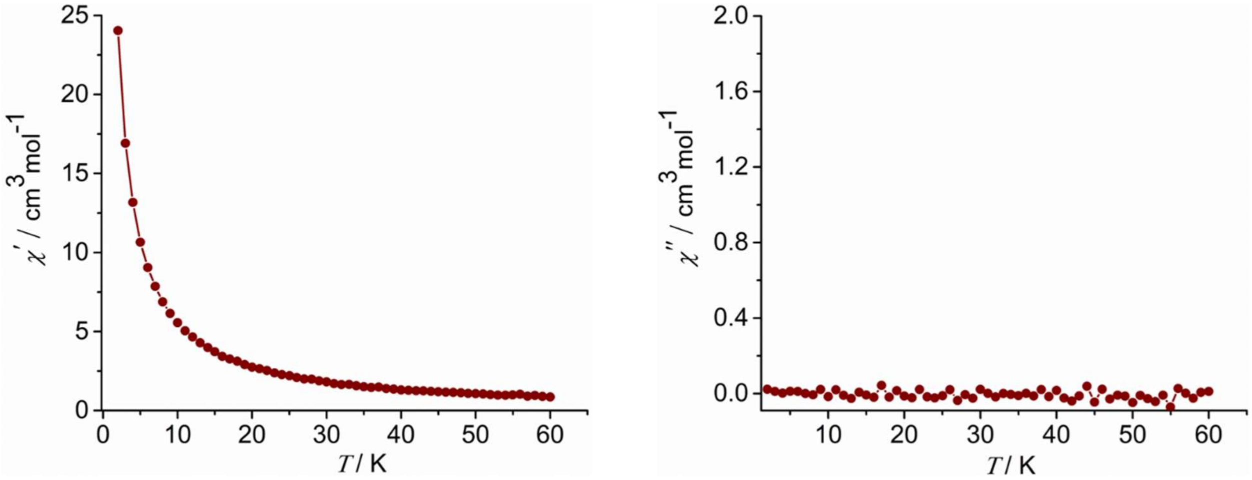

Figure S24. Real (left) and imaginary (right) components of the AC susceptibility as a function of temperature at 1000 Hz in an AC field of 3 Oe and zero DC field for $\mathbf{3_{Dy}}$.

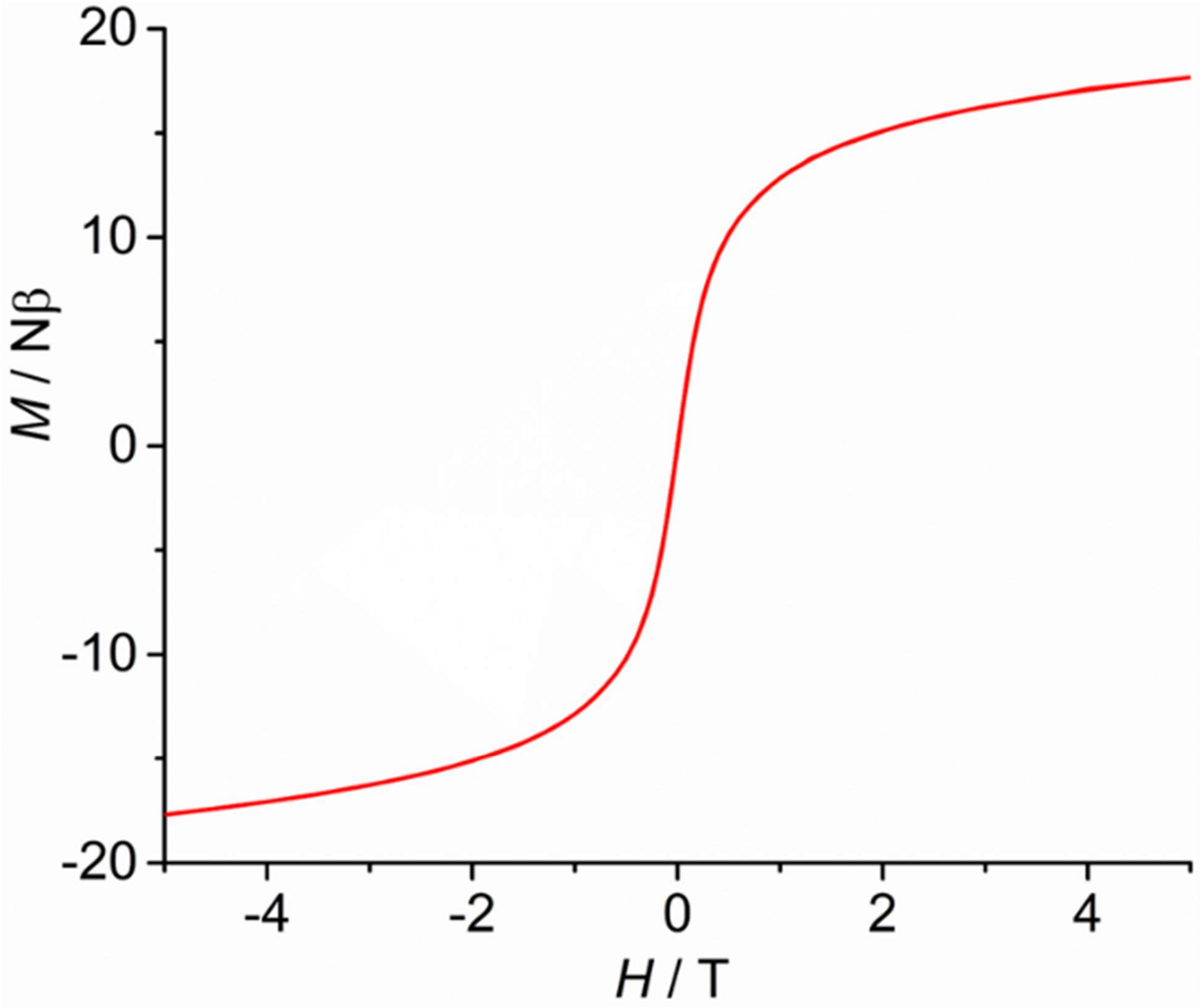

Figure S25. Magnetic hysteresis plot for $\mathbf{3}_{\mathbf{Dy}}$. The data were continuously collected at 1.9 K under the following field sweep rates: 5 mT s^{-1} |0-0.02| T; 10 mT s^{-1} |0.02-0.1| T; 50 mT s^{-1} |0.1-0.40| T; 100 mT s^{-1} |0.4-2.0| T; 200 mT s^{-1} |2.0-3.0| T; 500 mT s^{-1} |3.0-5.0| T.

To quantify the exchange interaction between gadolinium and the $[H_6HAN]^{3-}$ and $[Me_6HAN]^{3-}$ radical anion ligands (S = 1/2) in **2$_{Gd}$** and **3$_{Gd}$,** respectively, we performed a simulation using an isotropic Hamiltonian with an intermolecular term (zJ') as expressed in equation 1.

$$\hat{H} = -2J(S_{\mathrm{rad}} \cdot S_{\mathrm{Gd1}} + S_{\mathrm{rad}} \cdot S_{\mathrm{Gd2}} + S_{\mathrm{rad}} \cdot S_{\mathrm{Gd3}}) + \beta(g_{\mathrm{Gd1}} \cdot S_{\mathrm{Gd1}} + g_{\mathrm{Gd2}} \cdot S_{\mathrm{Gd2}} + g_{\mathrm{Gd3}} \cdot S_{\mathrm{Gd3}} + g_{\mathrm{rad}} \cdot S_{\mathrm{rad}}) \cdot B \quad (1)$$

Table S8. The parameters used to simulate susceptibility and magnetization data.

**Complex**	***J* / cm^{-1}**	***g***	***zJ'* / cm^{-1}**
**2$_{Gd}$**	+2.87 ± 0.03	g_{rad} = 2.0<br>g_{Gd} = 2.0	-0.002 ± 0.00004
**3$_{Gd}$**	+3.07 ± 0.04	g_{rad} = 2.0<br>g_{Gd} = 2.0	-0.001 ± 0.00004

DFT Calculations on 3_Y, 2_{Gd} and 3_{Gd}

Calculations were performed on the coordinates obtained from the X-ray structure using the ORCA 6.0.0. software package.[8] The position of hydrogen atoms was optimized at the DFT level using pure GGA PBE exchange-correlation function, keeping constant the position of other atoms. The BP86 functional was used,[19] and relativistic effects were included with the Douglas-Kroll-Hess Hamiltonian (DKH), together with the scalar relativistic contracted version of the basis functions def2-QZVP for Gd, and def2-TZVP for Y, C and H atoms.[20,21] The SARC/J auxiliary basis set[13] and RIJCOSX approximation[17,22] with a TightSCF convergence threshold were also used throughout the calculations. The NBO analysis was performed using the B3LYP/def2-TZVP/TZVP level of theory in Gaussian 16,[23] with the relativistic effective core potential (ECP) applied to Gd.[24]

Table S9. Energy and <S^2> spin expectation values for 3_Y calculated at the BP86/def2-TZVP level of theory.

**Energy (Hartree)**		**<S^2>**	
Doublet	Quartet	Doublet	Quartet
-15637.310373592	-15637.29873026	0.769087	3.756524

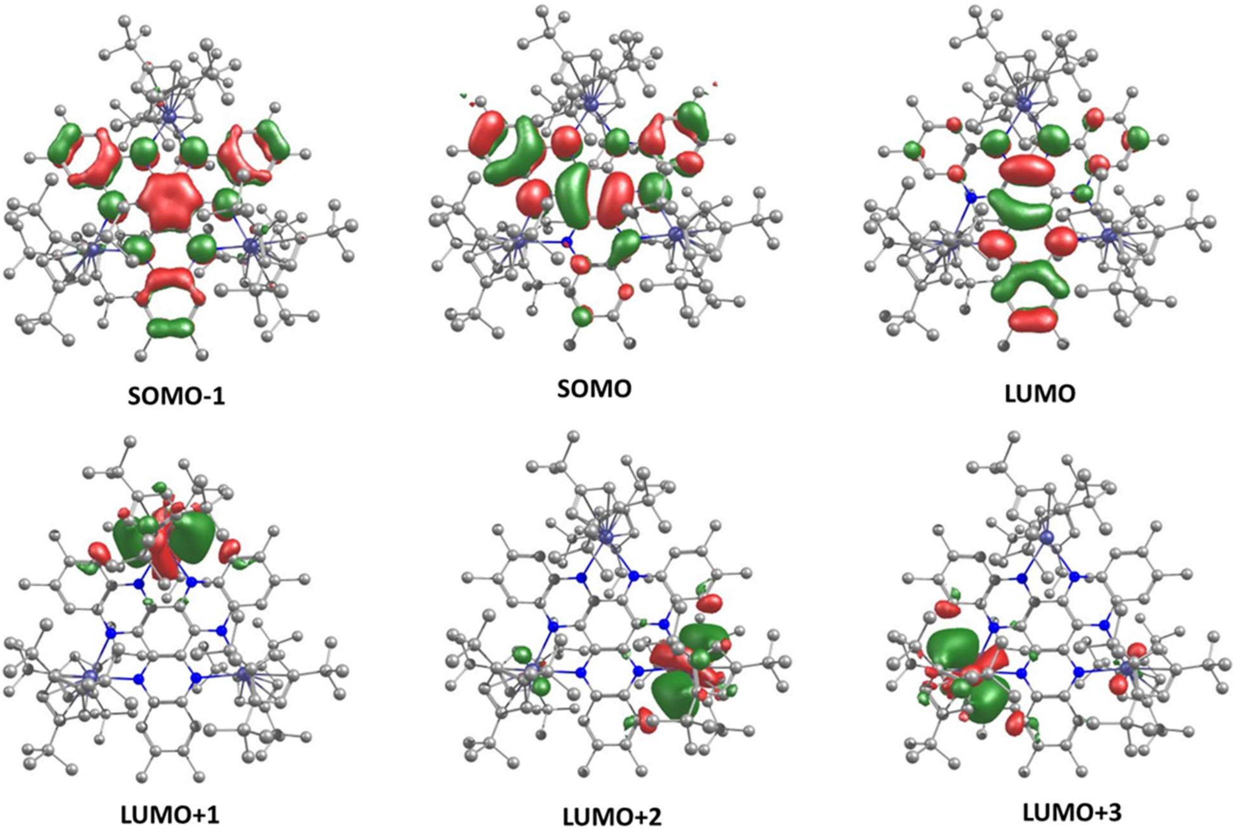

Figure S26. Frontier molecular orbitals in 3_Y (isosurface value = 0.027 a.u.).

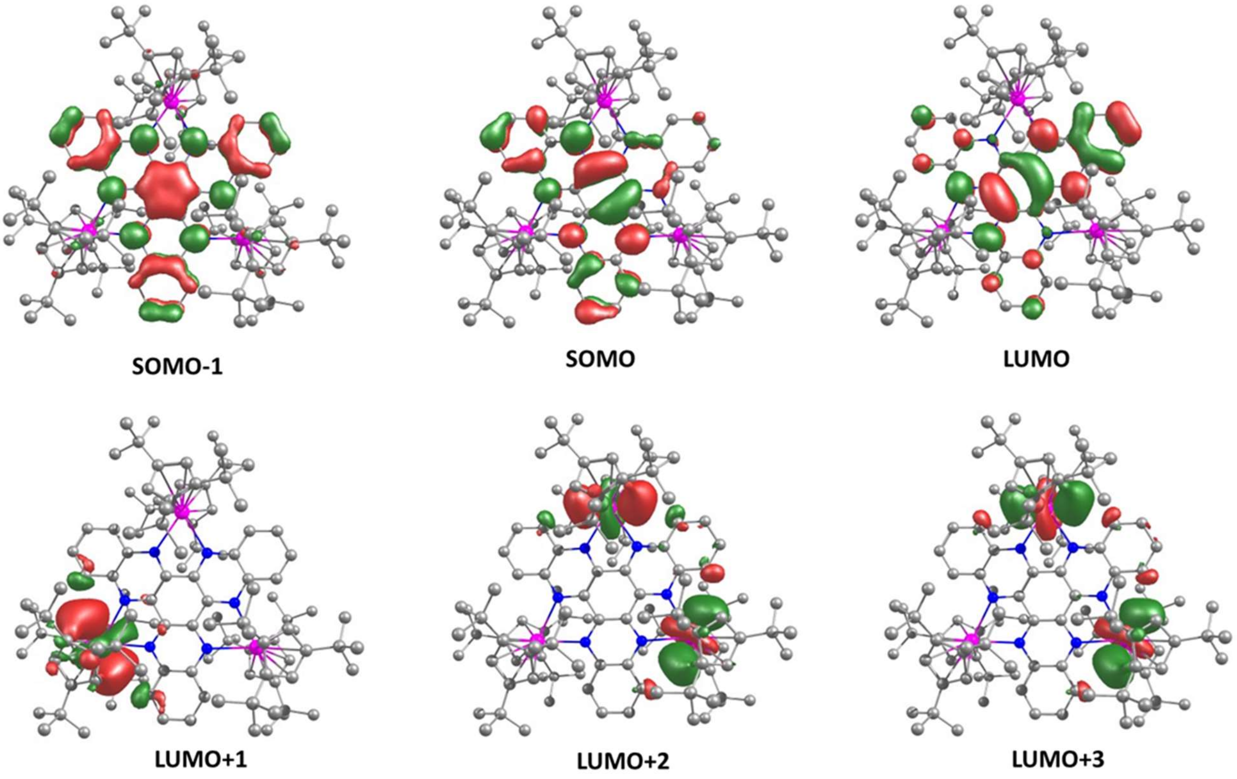

Figure S27. Frontier molecular orbitals in $\mathbf{2}_{\mathbf{Gd}}$ (isosurface value = 0.027 a.u.).

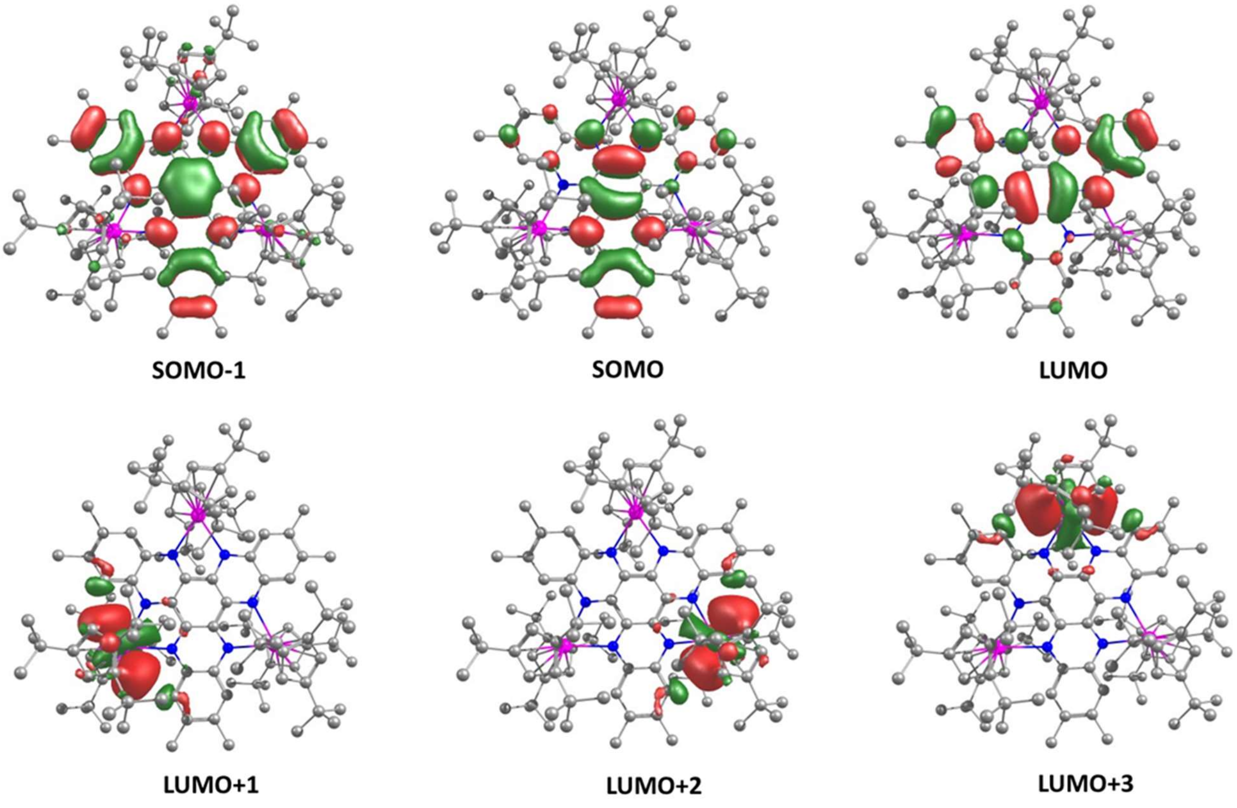

Figure S28. Frontier molecular orbitals in $\mathbf{3}_{\mathbf{Gd}}$ (isosurface value = 0.027 a.u.).

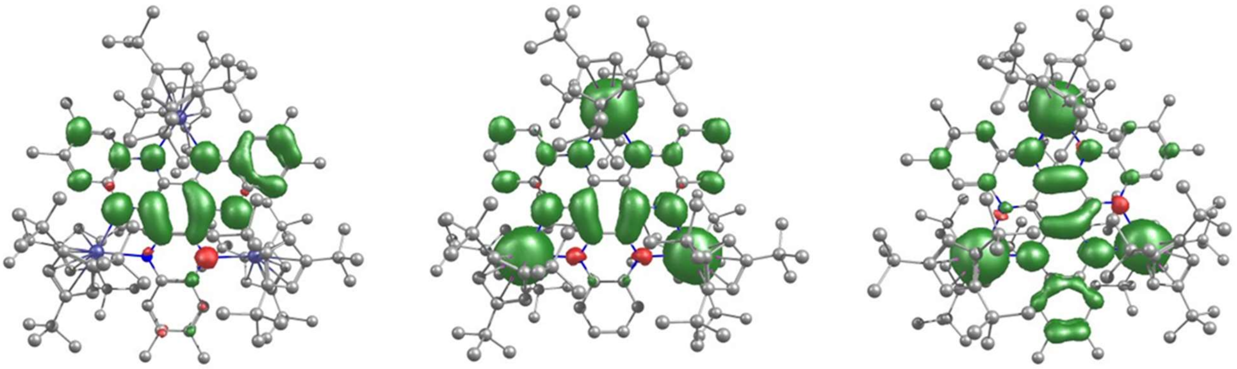

Figure S29. Spin density plots for $\mathbf{3_Y}$ (left), $\mathbf{2_{Gd}}$ (middle) and $\mathbf{3_{Gd}}$ (right) complexes (isosurface value = 0.0015 a.u.). Green and red shading indicate positive and negative spin densities, respectively.

Table S10. DFT calculated spin densities of selected atoms in $\mathbf{3_Y}$, $\mathbf{2_{Gd}}$ and $\mathbf{3_{Gd}}$.

**Atom**	$\mathbf{3_Y}$	$\mathbf{2_{Gd}}$	$\mathbf{3_{Gd}}$
Y, Gd	0.013543, 0.016819, 0.015205	7.104376, 7.104363 7.107500	7.103834, 7.107079 7.098927
N	0.056520	0.068817	0.086923
N	-0.043763	-0.034725	0.023787
N	0.130410	0.161917	-0.040604
N	0.186686	0.068803	0.181797
N	0.149796	-0.034737	0.150484
N	-0.007994	0.161890	-0.011063
C (central ring)	0.080915	-0.011310	0.101756
C (central ring)	0.010551	0.044154	0.081497
C (central ring)	-0.019204	0.097252	0.017433
C (central ring)	0.008426	-0.011302	0.050802
C (central ring)	0.049164	0.044148	0.028841
C (central ring)	0.109914	0.097248	-0.016538

Table S11. Second order perturbation theory NBO analysis on $\mathbf{2}_{Gd}$.

Donor NBO	Acceptor NBO	CT interaction / kJ mol^{-1}
N (s= 31.28%, p= 68.71%)	Gd (s= 0.09%, p= 52.23%, d= 47.51%, f= 0.17%)	55.77
N (s= 30.52%, p= 69.47%)	Gd (s= 2.03%, p= 46.32%, d= 51.28%, f= 0.37%)	48.83
N (s= 32.23%, p= 69.69%)	Gd (s= 52.23%, p= 20.23%, d= 26.22%, f= 1.32%)	33.18
N (s= 32.23%, p= 69.69%)	Gd (s= 0.21%, p= 15.72%, d= 71.45%, f= 12.62%)	35.77
N (s= 30.30%, p= 69.69%)	Gd (s= 52.23%, p= 20.23%, d= 26.22%, f= 1.32%)	38.53
N (s= 30.30%, p= 69.69%)	Gd (s= 0.21%, p= 15.72%, d= 71.45%, f= 12.62%)	30.42
N (s= 30.91%, p= 69.08%)	Gd (s= 40.83%, p= 47.62%, d= 11.50%, f= 0.05%)	44.89
N (s= 30.91%, p= 69.08%)	Gd (s= 0.00%, p= 48.82%, d= 50.79%, f= 0.39%)	34.06
N (s= 30.52%, p= 69.47%)	Gd (s= 40.52%, p= 46.68%, d= 12.74%, f= 0.06%)	31.17
N (s= 31.28%, p= 68.71%)	Gd (s= 2.03%, p= 46.32%, d= 51.28%, f= 0.37%)	19.28

Table S12. Second order perturbation theory NBO analysis on $\mathbf{3}_{Gd}$.

Donor NBO	Acceptor NBO	CT interaction / kJ mol^{-1}
N (s= 30.03%, p= 69.96%)	Gd (s= 40.48%, p= 46.44%, d= 13.03%, f= 0.05%)	47.24
N (s= 30.03%, p= 69.96%)	Gd (s= 0.77%, p= 47.51%, d= 51.34%, f= 0.38%)	26.61
N (s= 31.30%, p= 68.69%)	Gd (s= 40.48%, p= 46.44%, d= 13.03%, f= 0.05%)	37.61
N (s= 31.30%, p= 68.69%)	Gd (s= 0.77%, p= 47.51%, d= 51.34%, f= 0.38%)	41.59
N (s= 29.81%, p= 70.18%)	Gd (s= 43.97%, p= 44.30%, d= 11.68%, f= 0.05%)	41.97
N (s= 29.81%, p= 70.18%)	Gd (s= 0.02%, p= 44.40%, d= 55.19%, f= 0.40%)	30.84
N (s= 34.30%, p= 65.70%)	Gd (s= 40.13%, p= 47.46%, d= 12.37%, f= 0.05%)	45.23
N (s= 34.30%, p= 65.70%)	Gd (s= 0.07%, p= 55.03%, d= 44.56%, f= 0.34%)	35.06
N (s= 31.92%, p= 68.07%)	Gd (s= 40.13%, p= 47.46%, d= 12.37%, f= 0.05%)	44.39
N (s= 31.92%, p= 68.07%)	Gd (s= 0.07%, p= 55.03%, d= 44.56%, f= 0.34%)	33.81

References

1. Cendrowski-Guillaume, S. M.; Le Gland, G.; Nierlich, M.; Ephritikhine, M. Lanthanide Borohydrides as Precursors to Organometallic Compounds. Mono(cyclooctatetraenyl) Neodymium Complexes. *Organometallics* **2000,** *19*, 5654-5660.
2. Venier, C. G.; Casserly, E. W. Di-Tert-Butylcyclopentadiene and Tri-Tert-Butylcyclopentadiene. *J. Am. Chem. Soc.* **1990,** *112*, 2808-2809.
3. Goodwin, C. A. P.; Reta, D.; Ortu, F.; Liu, J.; Chilton, N. F.; Mills, D. P. Terbocenium: Completing a Heavy Lanthanide Metallocenium Cation Family with an Alternative Anion Abstraction Strategy. *Chem. Commun.* **2018,** *54*, 9182-9185.
4. Mondal, A.; Price, C. G. T.; Tang, J.; Layfield, R. A. Targeted Synthesis of End-On Dinitrogen-Bridged Lanthanide Metallocenes and Their Reactivity as Divalent Synthons. *J. Am. Chem. Soc.* **2023,** *145*, 20121-20131.
5. Barlow, S.; Zhang, Q.; Kaafarani, B. R.; Risko, C.; Amy, F.; Chan, C. K.; Domercq, B.; Starikova, Z. A.; Antipin, M. Y.; Timofeeva, T. V.; Kippelen, B.; Brédas, J.-L.; Kahn, A.; Marder, S. R. Synthesis, Ionisation Potentials and Electron Affinities of Hexaazatrinaphthylene Derivatives. *Chem. Eur. J.* **2007,** *13*, 3537-3547.
6. Dolomanov, O. V.; Bourhis, L. J.; Gildea, R. J.; Howard, J. A. K.; Puschmann, H. OLEX2: A Complete Structure Solution, Refinement and Analysis Program. *J. Appl. Cryst.* **2009,** *42*, 339-341.
7. Sheldrick, G. Crystal Structure Refinement with SHELXL. *Acta Cryst. C* **2015,** *71*, 3-8.
8. Neese, F. Software update: The ORCA Program System—Version 5.0. *WIREs Comput. Mol. Sci.* **2022,** *12*, e1606.
9. Perdew, J. P.; Burke, K.; Ernzerhof, M. Generalized Gradient Approximation Made Simple. *Phys. Rev. Lett.* **1996,** *77*, 3865-3868.
10. Perdew, J. P.; Burke, K.; Ernzerhof, M. Generalized Gradient Approximation Made Simple [Phys. Rev. Lett. 77, 3865 (1996)]. *Phys. Rev. Lett.* **1997,** *78*, 1396-1396.
11. Yanai, T.; Tew, D. P.; Handy, N. C. A New Hybrid Exchange–Correlation Functional Using the Coulomb-Attenuating Method (CAM-B3LYP). *Chem. Phys. Lett.* **2004,** *393*, 51-57.
12. van Wüllen, C. Molecular Density Functional Calculations in the Regular Relativistic Approximation: Method, Application to Coinage Metal Diatomics, Hydrides, Fluorides and Chlorides, and Comparison with First-Order Relativistic Calculations. *J. Chem. Phys.* **1998,** *109*, 392-399.
13. Rolfes, J. D.; Neese, F.; Pantazis, D. A. All-Electron Scalar Relativistic Basis Sets for the Elements Rb–Xe. *J. Comput. Chem.* **2020,** *41*, 1842-1849.
14. Izsák, R.; Neese, F. An Overlap Fitted Chain of Spheres Exchange Method. *J. Chem. Phys.* **2011,** *135*, 144105.
15. Neese, F.; Wennmohs, F.; Hansen, A.; Becker, U. Efficient, Approximate and Parallel Hartree–Fock and Hybrid DFT Calculations. A 'Chain-of-Spheres' Algorithm for the Hartree–Fock Exchange. *J. Chem. Phys.* **2009,** *356*, 98-109.
16. Barone, V.; Cossi, M. Quantum Calculation of Molecular Energies and Energy Gradients in Solution by a Conductor Solvent Model. *J. Phys. Chem. A* **1998,** *102*, 1995-2001.
17. Cossi, M.; Rega, N.; Scalmani, G.; Barone, V. Energies, Structures, and Eectronic Properties of Molecules in Solution with the C-PCM Solvation Model. *J. Comput. Chem.* **2003,** *24*, 669-681.
18. Bain, G. A.; Berry, J. F. Diamagnetic Corrections and Pascal's Constants. *J. Chem. Ed.* **2008,** *85*, 532.
19. Becke, A. D. Density-Functional Exchange-Energy Approximation with Correct Asymptotic Behavior. *Phys. Rev. A* **1988,** *38*, 3098-3100.
20. Aravena, D.; Neese, F.; Pantazis, D. A. Improved Segmented All-Electron Relativistically Contracted Basis Sets for the Lanthanides. *J. Chem. Theory Comput.* **2016,** *12*, 1148-1156.
21. Chmela, J.; and Harding, M. E., Optimized Auxiliary Basis Sets for Density Fitted Post-Hartree–Fock Calculations of Lanthanide Containing Molecules. *Mol. Phys.* **2018,** *116*, 1523-1538.
22. Izsák, R.; Neese, F., An Overlap Fitted Chain of Spheres Exchange Method. *J. Chem. Phys.* **2011,** *135*.
23. M. J. Frisch, G. W. Trucks, H. B. Schlegel, G. E. Scuseria, M. A. Robb, J. R. Cheeseman, G. Scalmani, V. Barone, G. A. Petersson, H. Nakatsuji, X. Li, M. Caricato, A. V. Marenich, J. Bloino, B. G. Janesko, R.

Gomperts, B. Mennucci, H. P. Hratchian, J. V. Ortiz, A. F. Izmaylov, J. L. Sonnenberg, D. Williams-Young, F. Ding, F. Lipparini, F. Egidi, J. Goings, B. Peng, A. Petrone, T. Henderson, D. Ranasinghe, V. G. Zakrzewski, J. Gao, N. Rega, G. Zheng, W. Liang, M. Hada, M. Ehara, K. Toyota, R. Fukuda, J. Hasegawa, M. Ishida, T. Nakajima, Y. Honda, O. Kitao, H. Nakai, T. Vreven, K. Throssell, J. A. Montgomery Jr. J. E. Peralta, F. Ogliaro, M. J. Bearpark, J. J. Heyd, E. N. Brothers, K. N. Kudin, V. N. Staroverov, T. A. Keith, R. Kobayashi, J. Normand, K. Raghavachari, A. P. Rendell, J. C. Burant, S. S. Iyengar, J. Tomasi, M. Cossi, J. M. Millam, M. Klene, C. Adamo, R. Cammi, J. W. Ochterski, R. L. Martin, K. Morokuma, O. Farkas, J. B. Foresman, D. J. Fox, 2016.
24. Cundari, T. R.; Stevens, W. J., Effective Core Potential Methods for the Lanthanides. *J. Chem. Phys.* **1993,** *98*, 5555-5565.
